# Structural Phylogenetics with Confidence

**DOI:** 10.1093/molbev/msaa100

**Published:** 2020-04-17

**Authors:** Ashar J Malik, Anthony M Poole, Jane R Allison

**Affiliations:** m1 Centre for Theoretical Chemistry and Physics, School of Natural and Computational Sciences, Massey University Auckland, Auckland, New Zealand; m2 Bioinformatics Institute, Agency for Science, Technology and Research, Singapore; m3 Bioinformatics Institute, School of Biological Sciences, University of Auckland, Auckland, New Zealand; m4 Digital Life Institute, University of Auckland, Auckland, New Zealand; m5 Biomolecular Interaction Centre, University of Canterbury, Christchurch, New Zealand; m6 Maurice Wilkins Centre for Molecular Biodiscovery, University of Auckland, Auckland, New Zealand

**Keywords:** phylogenetics, deep evolution, protein structure

## Abstract

For evaluating the deepest evolutionary relationships among proteins, sequence similarity is too low for application of sequence-based homology search or phylogenetic methods. In such cases, comparison of protein structures, which are often better conserved than sequences, may provide an alternative means of uncovering deep evolutionary signal. Although major protein structure databases such as SCOP and CATH hierarchically group protein structures, they do not describe the specific evolutionary relationships within a hierarchical level. Structural phylogenies have the potential to fill this gap. However, it is difficult to assess evolutionary relationships derived from structural phylogenies without some means of assessing confidence in such trees. We therefore address two shortcomings in the application of structural data to deep phylogeny. First, we examine whether phylogenies derived from pairwise structural comparisons are sensitive to differences in protein length and shape. We find that structural phylogenetics is best employed where structures have very similar lengths, and that shape fluctuations generated during molecular dynamics simulations impact pairwise comparisons, but not so drastically as to eliminate evolutionary signal. Second, we address the absence of statistical support for structural phylogeny. We present a method for assessing confidence in a structural phylogeny using shape fluctuations generated via molecular dynamics or Monte Carlo simulations of proteins. Our approach will aid the evolutionary reconstruction of relationships across structurally defined protein superfamilies. With the Protein Data Bank now containing in excess of 158,000 entries (December 2019), we predict that structural phylogenetics will become a useful tool for ordering the protein universe.

## Introduction

**Fig. 1.**
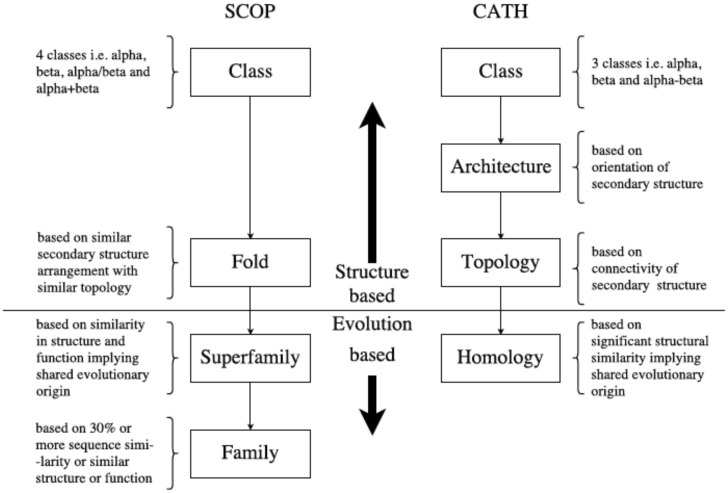
Organization of SCOP and CATH databases: SCOP (Andreeva et al. 2019) arranges protein structures into classes, Folds, Superfamilies, and Families. CATH (Sillitoe et al. 2019) uses Classes, Architectures, Topologies, and Homologies to organize protein structures. The horizontal split marks a boundary which separates structure- and evolution-based groupings. Structures grouped together in Homology (CATH) and Family and Superfamily (SCOP) likely share a common evolutionary origin.

Structure appears in general to be better conserved than sequence (Illergård et al. 2009). It also forms the basis for grouping the universe of proteins into superfamilies, many of which traverse the “twilight zone,” across which sequences are too dissimilar for reliable homolog identification (Rost 1999). Two widely used databases, SCOP (Andreeva et al. 2019) and CATH (Sillitoe et al. 2019), have ordered the protein universe in a hierarchical manner based on secondary structure organization and evolutionary origin (fig. 1). These hierarchies thus include both higher-level groupings (e.g., Class) based on similar but nonevolutionary structural characteristics (such as being composed of α-helices) and lower-level groupings such as “Homology” (CATH) or “Superfamily” (SCOP) (fig. 1), where constituent proteins share structural similarities suggestive of descent from a common ancestor. However, being hierarchical, neither database provides specific information on the evolutionary relationships between members at a structurally defined level; relationships between entities within a hierarchical level are left as unresolved polytomies.

Phylogenetic analysis of superfamilies can provide this missing detail. Some groups have successfully employed hybrid sequence-structure approaches to construct structure-informed pairwise sequence alignments (Challis and Schmidler 2012), or Bayesian phylogenetics with a joint model of sequence and structure (Herman et al. 2014). However, in many cases, superfamilies are united by structural similarity but lack sufficient sequence-level similarity for conventional phylogenetic analysis based on multiple sequence alignments. In cases where there is evidence for common descent, but insufficient sequence similarity for sequence-based phylogeny, phylogenies can be generated from structural data alone (Bujnicki 2000; Breitling et al. 2001; Garau et al. 2005; Lundin et al. 2012, 2015). In such cases, the atom-positional root mean-squared deviation (RMSD) (or measures derived from the RMSD) between protein structures may be used together with distance-based methods to reconstruct structural phylogenies, thereby tracing the deep evolutionary relationships of protein structures sharing a common origin.

For protein structural phylogenetics, the structural comparison metric, the inference method, and the assessment of the statistical significance of the inferred relationships all present challenges. Numerous protein structure comparison metrics exist, such as those implemented in DALI (Holm and Sander 1995), CE (Shindyalov and Bourne 1998), TM-Align (Zhang and Skolnick 2005), and SSM (Krissinel and Henrick 2004), each of which uses a different algorithm for calculating structural similarity. Distance-based phylogenies have been successfully employed in structural phylogenetics in lieu of theoretical or empirically derived models of structural mutation, and the use of tools such as splitstree (Huson and Bryant 2006) has allowed assessment of the tree-likeness of structural phylogenies (Lundin et al. 2012, 2015). However, there is currently no means of assessing the statistical significance of the resulting trees or networks. The sequence-based bootstrap method cannot be adapted for use with protein structures, not least because the underlying assumption made for the sequence data, namely that characters are independent and identically distributed, does not apply to protein structure. Phylogenies inferred from structure data can be assessed by examining congruence with trees derived from sequence data, however, this is limited to assessing evolutionary signal in shallower nodes where sequence-based phylogenies can be generated (Lundin et al. 2012), and is noninformative for the deeper nodes, which can only be recovered using structural data. Finally, it is in principle possible to overlay characters as a means of providing some qualitative assessment of the inferred tree. However, it can be difficult to assess the stability of characters such as dimer interfaces, which can alter during evolution (Devenish and Gerrard 2009; Griffin et al. 2010; Allison et al. 2016), so their evolution is most helpfully assessed in light of a tree (Lundin et al. 2012), rather than being used to establish relationships per se. For structural phylogenetics to fill the gap between hierarchical structural classification schemes and sequence-based phylogeny, it is thus critical that statistical methods equivalent to the bootstrap be developed.

To that end, we present a structural analog to the bootstrap that statistically gauges the robustness of structural phylogenetic relationships. We first assessed the utility of structural phylogenetic analysis using the *Q*_score_ comparison metric. We assessed the impact of protein length on this metric and find that the *Q*_score_ is best employed where structures have very similar (>90%) lengths. We next examined the impact of shape fluctuations generated during molecular dynamics (MD) simulations on pairwise structural comparisons. We find that simulations do impact shape, but not so drastically as to eliminate evolutionary signal. Buoyed by the latter result, we then show that the structural diversity generated during MD or Monte Carlo (MC) simulations can be harnessed to generate a measure of confidence for inferred structural phylogenies, similar to the sequence-based bootstrap. As a demonstration of the method, we assess the previously published structural phylogeny of the ferritin-like superfamily, a data set where sequence similarity is too low to reliably assess evolutionary relationships (Lundin et al. 2012).

## Results

### Use of the *Q*_score_ for Structural Phylogeny

Structure-based phylogenetics currently involves generating a set of pairwise structural comparisons from which distances can be derived, generating a distance matrix. Previous studies derived distances from the atom-positional RMSD or the quality score (*Q*_score_) calculated by the SSM tool (Krissinel and Henrick 2004). After testing a variety of metrics (Lundin et al. 2012; Malik 2018), we chose *Q*_score_, which is attractive as it allows for indels and includes both alignment quality (RMSD) and alignment length.

The *Q*_score_ metric compares the atomic positions of all α-carbon atoms from the *N*_align_ residues considered comparable by SSM. Reduction of secondary structure elements to vectors and rigid geometrical similarity between vectors, across structures, determines residues (*N*_align_) which are considered comparable. Protein structure comparisons using *Q*_score_ are not strictly commutative, but return highly similar results, with variations that are two to three orders of magnitude smaller than the total score when the order of the comparison is reversed. It generates a normalized score in the range [0,1] through inclusion of both the number of aligned residues and the total number of residues in each protein. As large *Q*_score_ values correspond to more similar structures, $1-Q_{\text{score}}$ is used as a measure of distance.

Despite these positive aspects, it is unclear how features of the data, such as length differences between proteins, might impact *Q*_score_. We therefore sought to assess the performance of *Q*_score_ for structural phylogeny.

A key feature of the *Q*_score_ metric is that the contributions made by protein size, including the length of each protein and the number of aligned residues (*Q*_length_, eq. 2), and by structural variations (*Q*_shape_, eq. 3), can be deconvoluted:

(1) $$Q_{\text{score}}=\frac{N_{\text{align}}^{2}}{[1+{\left(\frac{\text{RMSD}}{R_{0}}\right)}^{2}]N_{1}N_{2}},$$

(2) $$Q_{\text{length}}=\frac{N_{\text{align}}^{2}}{N_{1}N_{2}},$$

(3) $$Q_{\text{shape}}=\frac{1}{\left[1+{\left(\frac{\text{RMSD}}{R_{0}}\right)}^{2}\right]}.$$

We investigated the effect of *Q*_length_ and *Q*_shape_ on the structural phylogenies produced, and examined the thresholds in the protein length differences and structural dissimilarity beyond which the *Q*_score_ is unlikely to be informative.

### Effect of Differing Protein Length on *Q*_score_

To investigate the effect of protein length difference (*Q*_length_) on *Q*_score_ values generated from pairwise comparisons, we investigated three SCOP superfamilies with different length distributions (the globin, trypsin-like serine proteases, and aldo-keto reductase [NADP] superfamilies), whose lengths fall in the typical ranges for single protein domains in the Protein Data Bank (PDB) (supplementary table S1 and fig. S1, Supplementary Material online).

Each protein from these three data sets was decomposed into length fractions, with each fraction starting from the N-terminal region of the protein and increasing by 10% of the total number of residues in the protein relative to the previous fraction (supplementary fig. S2, Supplementary Material online). The structure of each fraction is identical to the respective structural fragment in the complete structure. In this way, the effect of structural variation is eliminated so that only contributions due to differences in the total number of residues, *N*_1_, *N*_2_, and in the number of aligned residues, *N*_align_, are considered.

**Fig. 2.**
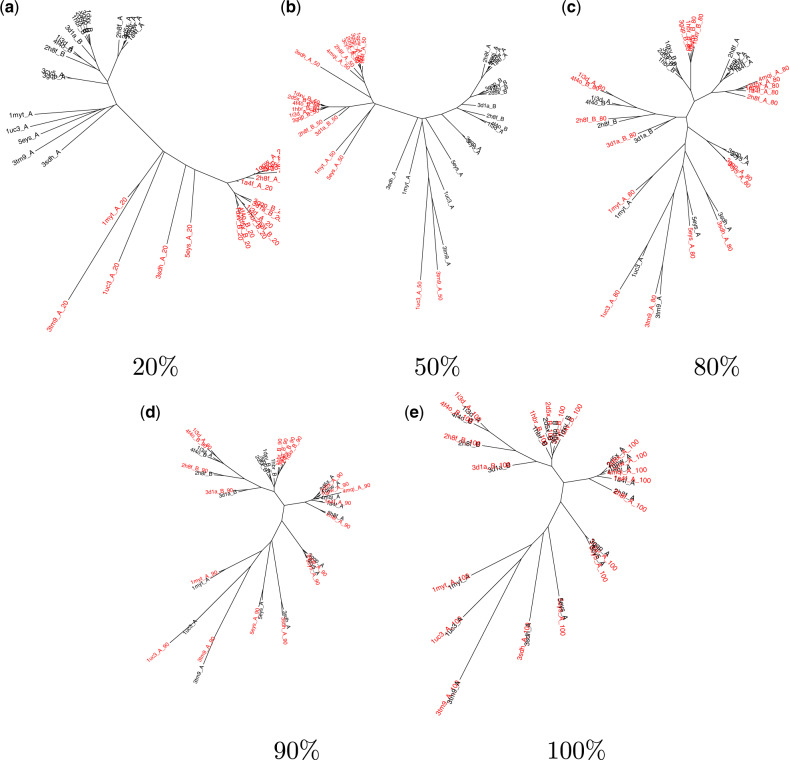
Phylogenetic trees for proteins from the globin family built using structural data sets that comprise the indicated fraction of each structure (red) together with the complete structures (black). Only trees built from five of the fractional structural data sets are shown here; enlarged versions of all ten are provided in supplementary figures S3–S12, Supplementary Material online. (*a* and *b*) For fractional structures comprising up to 70% of the complete structure, fraction size dominates the tree structure. Clade groupings are sometimes reproduced for fractional structures comprising 70% to (*c*) 80% of each protein, but residual differences to (*e*) the true tree remain even for (*d*) 90% fractional structures.

**Fig. 3.**
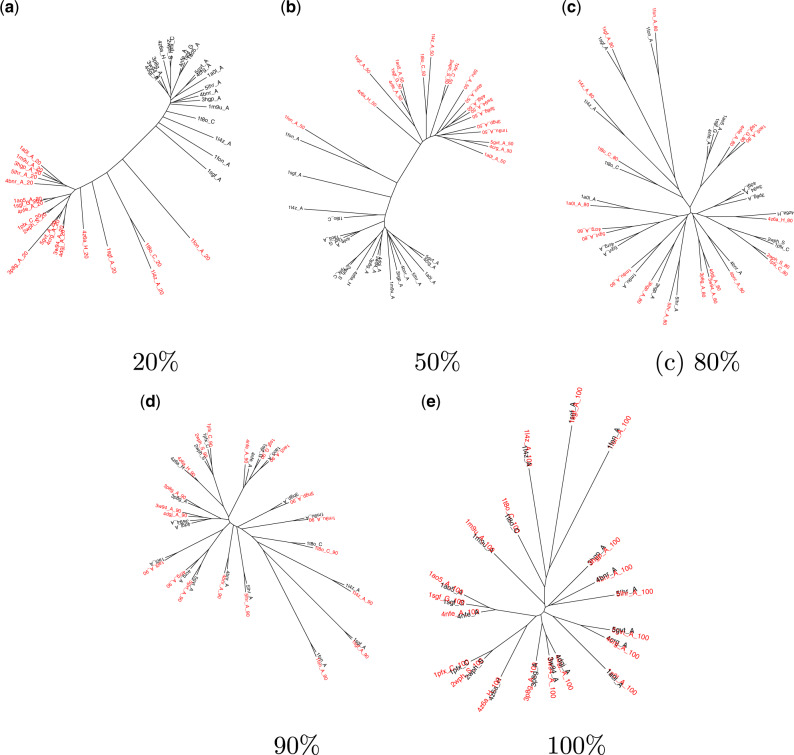
Phylogenetic trees for proteins from the trypsin-like serine protease family built using structural data sets that comprise the indicated fraction of each structure (red) together with the complete structures (black). Only trees built from five of the fractional structural data sets are shown here; enlarged versions of all ten are provided in supplementary figures S13–S22, Supplementary Material online. (*a*) For fractional structures comprising up to 50% of the complete structure, fraction size dominates the tree structure. Clade groupings are sometimes reproduced for fractional structures comprising (*b*) 50% to (*c*) 80% of each protein, but residual differences to (*e*) the true tree remain even for (*d*) 90% fractional structures.

**Fig. 4.**
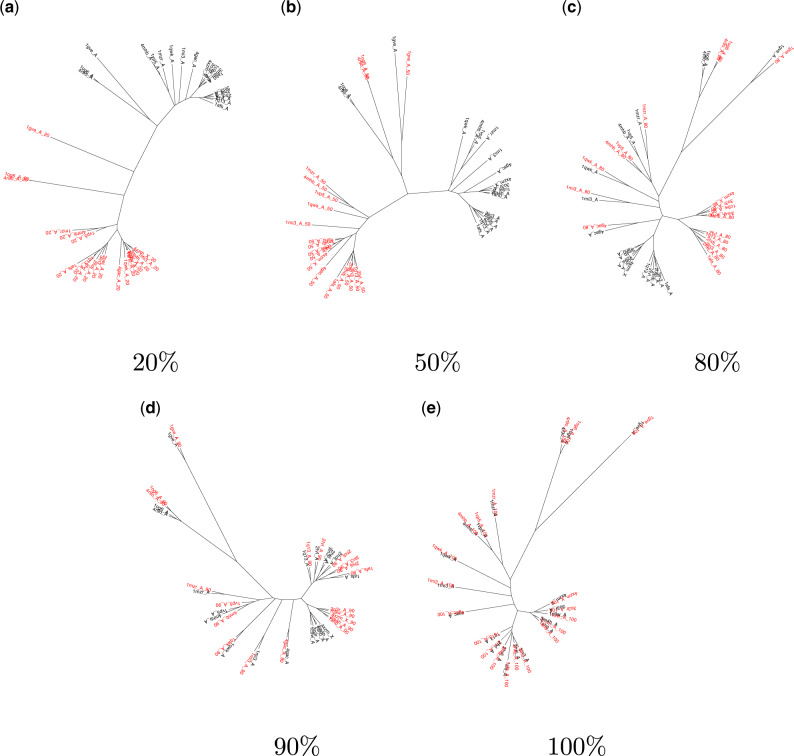
Phylogenetic trees for proteins from the aldo-keto reductase (NADP) family built using structural data sets that comprise the indicated fraction of each structure (red) together with the complete structures (black). Only trees built from five of the fractional structural data sets are shown here; enlarged versions of all ten are provided in supplementary figures S23–S32, Supplementary Material online. (*a*) For fractional structures comprising up to 50% of the complete structure, fraction size dominates the tree structure. Clade groupings are sometimes reproduced for fractional structures comprising (*b*) 50% to (*c*) 80% of each protein, but residual differences to (*e*) the true tree remain even for (*d*) 90% fractional structures.

Distance trees were generated from the *Q*_length_ values for each fractional data set plus the set of full-length structures. The “true” tree, $T_{100\%}$, is considered recovered if each fractional structure has the same relationships as its complete structural counterpart. As is clear from figures 2–4, heavily truncated structures group separately to the complete structures for all three families. Although fractional structures comprising 40–70% of the complete structure sometimes occupy the same clade as the complete structure, there are nevertheless residual differences even when the fractional structures comprise 90% of the complete structure. Although it is not surprising that the most truncated structures do not group with the complete structures on the trees, as *Q*_score_ relies on the alignment of secondary-structure elements, it is telling that in some cases, only very small length differences can impact placement on the tree.

**Fig. 5.**
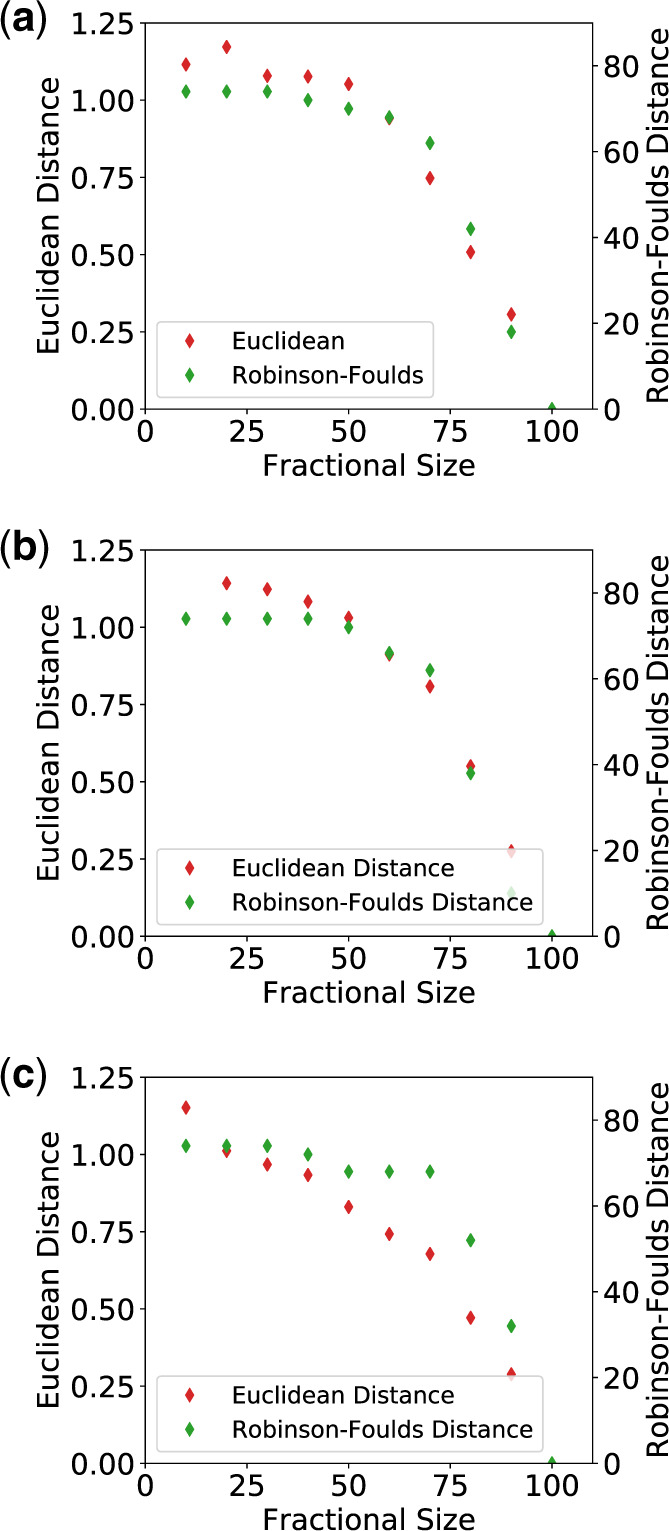
The Euclidean and Robinson–Foulds (Robinson and Foulds 1981) distances between fractional trees, $T_{10\%}$ through $T_{90\%}$, and the true tree, $T_{100\%}$ for the (*a*) globin, (*b*) trypsin-like serine protease, and (*c*) aldo-keto reductase (NADP) superfamilies. As the length difference between the complete and fractional structures decreases, the topologies of the fractional trees approach those of the true tree, $T_{100\%}$.

As the above assessment examines node placement qualitatively, we decided to examine differences between the trees quantitatively by measuring the Euclidean (equivalent to Felsenstein’s [2004] branch length distance) and Robinson–Foulds (Robinson and Foulds 1981) distances between each fractional tree ($T_{10\%}$ through $T_{90\%}$) and the “true” tree, ($T_{100\%}$) (fig. 5). In general, both distance measures decrease in concert with the length difference between the two structural data sets, that is, between the fractional and complete structures, and remain similarly high until the size of the fractional proteins reaches 60%, at which point both distance measures decrease toward zero. Not until fractional protein sizes of 90% do the distance measures become sufficiently low to indicate similar trees.

Our results indicate that the best resolution was achieved for fractions comprising between 90% and 100% of the complete structure(s). Although truncation of identical structures is not exactly equivalent to the comparison of evolutionarily related nonidentical structures of different length, it nevertheless permits us to examine how *Q*_score_ is impacted by length differences. In our test, we removed evolutionary signal through truncation, so are cautiously optimistic that, for real data, these cut-offs are more stringent than they may need to be. That said, it is clear that structures whose length varies by ≤10% can be confidently used for *Q*_score_-based analysis of evolutionary relationships. The length effect we observe suggests that, for data sets containing proteins with very different lengths, caution is warranted, and may impact the results, even where the length variation is modest. We recommend, therefore, that a similar truncation analysis is undertaken prior to using a *Q*_score_-based approach to structure comparison in order to assess the extent to which size variation affects the results.

### Effect of Protein Shape on *Q*_score_

The complexity of protein structure makes exploring the contribution of morphometric changes toward the *Q*_score_ (i.e., *Q*_shape_) a nontrivial problem. It is not possible to vary protein structure in such a controlled manner as length. Nevertheless, we sought to examine how variations in “shape” might impact structure-based phylogenies.

To examine the degree of shape perturbations that a protein can undergo without losing its structure, we performed MD simulations to sample alternative conformations of 53 proteins from the ferritin-like superfamily (supplementary table S2, Supplementary Material online). MD simulations allow a molecular structure to explore its structural neighborhood by allowing the atoms to move according to potential functions that account for their bonded and nonbonded interactions. This produces a set of conformations of each protein where the shape may change, but the length does not, hence any effect from protein length differences is excluded.

For each protein, the conformation from which the simulation was initiated was compared with the conformations sampled during the MD simulation by computing the Cα atom-positional RMSD (supplementary fig. S33*A*, Supplementary Material online). This was then used to compute the *Q*_shape_ score between each sampled conformation and the initial conformation (supplementary fig. S33*B*, Supplementary Material online). A *Q*_shape_ value of “1” indicates identical conformations, and the value will decrease as the compared structures diverge in conformation. RMSD shows the opposite behavior, increasing with the distance between structures, and is not bounded. The initial steep increase in the RMSD value occurs as the protein accumulates kinetic energy and adjusts to the simulation conditions, after which it samples conformations that are typically 1.6−6 Å from the initial conformation. The *Q*_shape_ values show an inverted trend, with plateau values of 0.2−0.8. A few proteins show particularly large RMSD values that fluctuate dramatically. Visual examination of their simulation trajectories revealed this to be due to large-scale motion of unstructured termini, and our later analyses (including a structural phylogeny of the ferritin-like superfamily) show that this does not appear to perturb our ability to correctly place these proteins on the phylogenetic network.

The deviation of the *Q*_shape_ values from 1.0 shows that this aspect of *Q*_score_ is sensitive to the relatively subtle changes in protein conformation sampled during a MD simulation. This is encouraging, as it suggests that MD simulations can be used confidently to introduce fluctuations in the structural data, which can be used to evaluate the robustness of the inferred evolutionary relationships between structures. However, it also serves as a warning. Protein molecules are highly dynamic, and thus subtle differences in the structures used to infer evolutionary relationships may result in vastly different tree topologies and hence lead to an alternative evolutionary interpretation. The question, therefore, is whether the degree of structural fluctuation that occurs during a MD simulation is sufficiently drastic as to eliminate evolutionary signal.

To answer this question, we sought to carry out an analogous procedure to that used to test length effects. We determined the central structure of the simulated ensemble of each protein using RMSD-based conformational clustering, and plotted the range of Cα atom-positional RMSD values from the central structure (supplementary fig. S34, Supplementary Material online). These ranges have a variety of medians and widths, indicating that each protein undergoes a different degree of structural fluctuation during MD simulation. We used the central structures to build a reference tree. We then tried two different approaches to building trees from structures that differ by varying, but controlled, degrees from their corresponding central structure.

First, we took a parametric approach to sampling structures from the RMSD distributions, which aimed to overcome the different ranges of RMSD values in a similar manner to our sampling of fractions of the protein lengths (which vary between proteins) when testing the length contribution to *Q*_score_. For each protein, we ranked the RMSD values and divided them into ten bins, each of which contains 10% of the RMSD data. It is clear from the RMSD ranges (supplementary fig. S34, Supplementary Material online) that, for instance, the fifth-ranked bin for one protein might contain only small RMSD values, whereas for another, it might contain much larger RMSD values, depending on their flexibility. Regardless, for each bin, we randomly sampled a structure from each trajectory and used these to build a sample tree. We repeated this 1,000 times for each bin, and computed the Euclidean and Robinson–Foulds distance between each of the 1,000 sample tree and the reference tree (supplementary fig. S35*A*, Supplementary Material online). We see an increase in the distance between the sample tree and reference tree as the ranked RMSD bin number increases, although there is a lot of noise due to the variability in the magnitude of the RMSD values between different proteins.

Second, to avoid the problems with variation across different proteins in the magnitude of RMSD values for a given bin, we assigned the raw RMSD values to bins. We determined the total range of RMSD values across the simulated ensembles of all 53 proteins. We eliminated the upper part of this range as few proteins sampled the highest RMSD values, leaving a raw RMSD range of 0.5−5.5 Å, which we divided into ten raw RMSD bins of 0.5 Å width. The number of proteins with RMSD values in a bin decreases as the bin number (RMSD value) increases (supplementary table S3, Supplementary Material online). This presented us with two options regarding the reference tree to use. We could build a new reference tree for each bin, using only the central structures of the trajectories for which there is RMSD data in that bin. This means that the sample trees built for each bin will have the same number of taxa as their corresponding reference tree. Alternatively, we continue to use the global reference tree built from all 53 proteins. This means that for some bins, the sample trees will have fewer taxa than the reference tree. In the calculation of the Euclidean and Robinson–Foulds distances, the branch length for a tree that is missing that branch is set to zero, and hence the difference in the length of that branch will be maximal.

For each of these two options, for each bin, we randomly sampled a structure of each protein for which there was RMSD data in that bin from its simulated ensemble and built a sample tree, which we compared with the reference tree. This was again repeated 1,000 times for each bin, and the Euclidean and Robinson–Foulds distance between each sample tree and the corresponding reference tree was computed (supplementary fig. S35*B* and *C*, Supplementary Material online).

When a different reference tree is used for each bin, the distance between the sample and reference trees initially increases as the RMSD increases. From bin 5 (RMSD values of 2.5−3.0 Å) onward, however, the distances appear to mirror the number of trajectories for which there are RMSD data in that bin (and for which the reference and sample trees were built). This likely reflects the inherent reduction in the distances between trees with fewer taxa and is therefore not a true reflection of the similarity of the reference and sample trees.

When the tree built from all 53 proteins is used as the reference tree for all bins, we see that as the RMSD increases and the number of contributing trajectories decreases, the distance between the overall reference tree and the current tree increases. Although this result is in line with the results of the parametric approach, it is important to note that the distance values include maximum contributions from the taxa for which there are no RMSD data in that bin.

Although none of these approaches is ideal, with each suffering from different problems, the same trend emerges throughout, namely that an increase in RMSD from the structures used to build the reference tree correlates with an increase in the distance between the reference and sample tree. Given the difficulty we encountered in designing this analysis, as well as the different degrees of flexibility of different proteins, it is not appropriate to provide a RMSD cut-off beyond which structural phylogenetics should not be attempted. We suggest, however, that tests such as these are applied to ascertain the sensitivity of the results to structural fluctuations prior to drawing biological conclusions.

### Assessing Statistical Significance of Phylogenetic Relationships Using MD

**Fig. 6.**
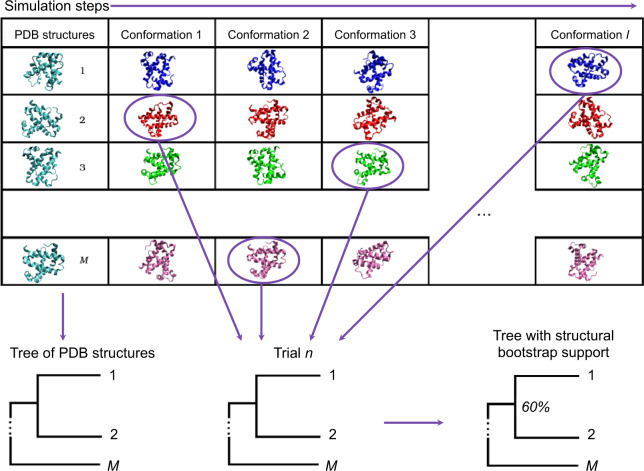
Overview of a bootstrap method for structure comparisons. An ensemble of possible conformations is generated for each of m∈M proteins using MD simulation. For each of n∈N trials, a conformation *c*_m_ is randomly selected from each of the *M* ensembles to populate a new trial data set *C*_n_. Pairwise comparison of the conformations in each trial data set *C*_n_ generates new distances from which a NJ tree *T*_n_ is created. Each trial tree, *T*_n_, is compared with the reference tree *T*_0_. If a relationship between structures in the reference tree *T*_0_ is recreated in the trial tree *T*_n_, it is counted. The nodes of *T*_0_ are labeled with the fraction of trial trees in which the relationship was recovered, providing a measure of the statistical support for that node.

Assessment of the statistical significance of structure-based phylogenies requires an analog to the bootstrap method used for protein sequences. The standard nonparametric sequence-based bootstrap method cannot be generalized to protein structures because it relies on the assumption that characters are independent and identically distributed, which does not apply to protein structure. We reasoned that resampling structure might be achieved through creating alternative conformations of the entire protein structure, determined through MD simulations. Selection of conformations at random from a pool of possible conformations of each protein allows a set of trial trees to be built, from which the statistical support for each node in the tree built from the original crystal structure data can be enumerated. The method is outlined and illustrated in figure 6.

Conformations are selected only from the “production” period of the simulation, that is, after the structure has equilibrated, as indicated by the plateau in the RMSD (Grossfield and Zuckerman 2009). Discarding this “burn-in” phase avoids biasing support in favor of the crystal structure tree, as conformations in this initial phase of the simulation will always be very similar to the initial structure. In keeping with the spirit of the conventional sequence bootstrap method, we select conformations from the remainder of the simulation at random.

### Trialing MD for Assigning Confidence to a Structural Phylogeny

We next tested our MD-based approach for assigning confidence to a structural phylogeny, providing an opportunity to determine whether the conformational sampling that occurs during a MD simulation destroys evolutionary signal. We chose two protein families, globins and the “ribonucleotide reductase (RNR)-like” subset of the Ferritin superfamily, which comprises proteins most closely related to the small subunit of class I RNRs (supplementary table S4, Supplementary Material online), because hemoglobins have diverged relatively recently, whereas the RNR-like proteins are more diverged and may thus be better suited for structural phylogenetics. The globins include α- and β-hemoglobins, which are known from sequence-based analysis to be the result of a relatively recent gene duplication and divergence event (Storz et al. 2013) and have highly similar structures (supplementary fig. S36, Supplementary Material online). The RNR-like proteins are a subset of the ferritin-like protein superfamily that are more diverged than the globins (Lundin et al. 2009, 2012), and hence have less similar structures (supplementary fig. S37, Supplementary Material online). Eight protein structures were selected from each family, including four α- and four β-hemoglobins, and MD simulations were undertaken.

**Fig. 7.**
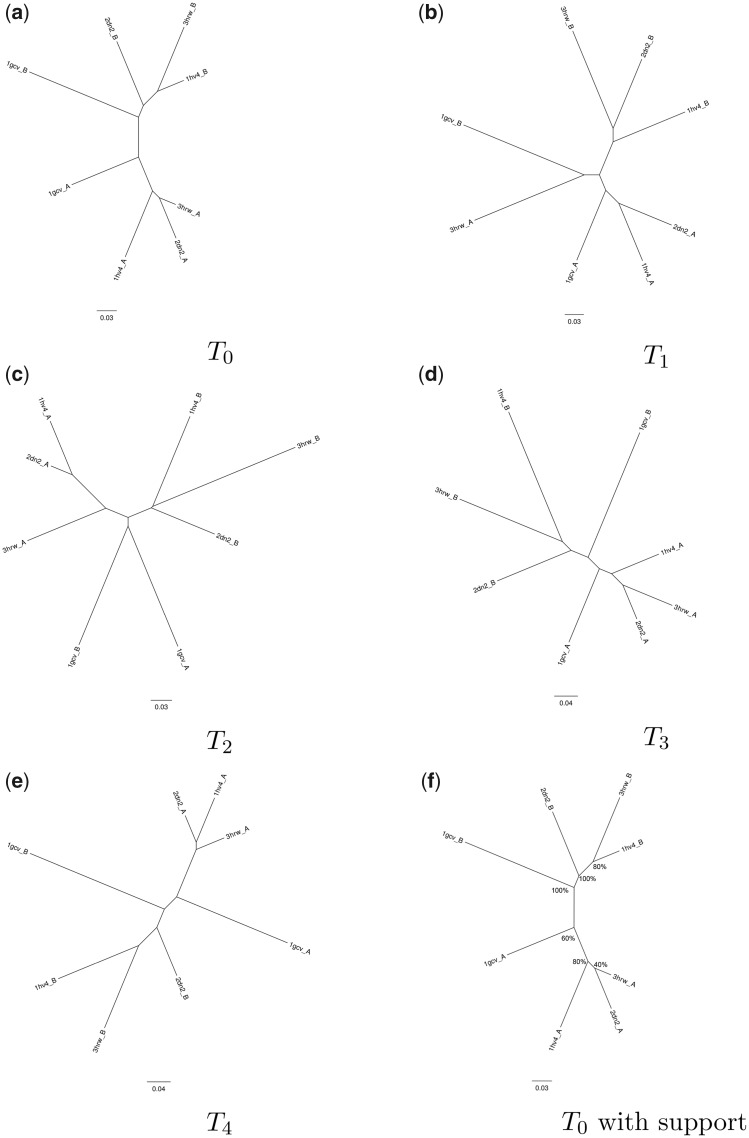
Illustration of MD-based bootstrap trials on structures from the globin family. The recent divergence of the α and β globin chains is reproduced with 100% confidence, but the relationships between the α chains have low support. The annotated tree (*f*) uses *T*_0_, the reference tree, and shows the relationships recovered as a percentage of the trials conducted (in this case, five, (*a*) *T*_0_ – (*e*) *T*_4_).

**Fig. 8.**
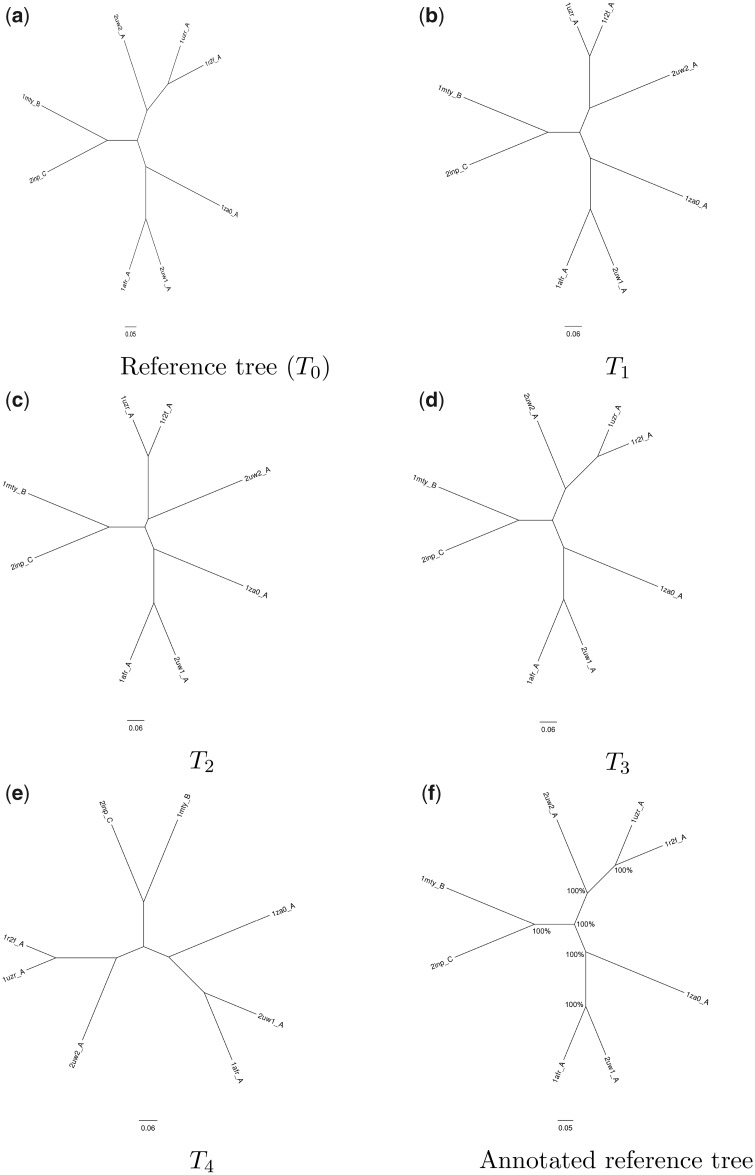
Illustration of MD-based bootstrap trials on structures from the ribonucleotide reductase-like family. All relationships have 100% support from this limited set of bootstrap trials. The annotated tree (*f*) uses *T*_0_, the reference tree, and shows the relationships recovered as a percentage of the trials conducted (in this case, five, (*a*) *T*_0_ –* (e) T*_4_).

As expected, only weak statistical support emerges when the structures are very similar, as is the case with globins (fig. 7). This is likely because the ensemble of alternative conformations of each protein structure sampled during the simulation overlaps with those of closely related structures; that is, the structural fluctuations that occur during the MD simulation obfuscate evolutionary signal in the structural data. That said, the divergence of the α and β globins was resolved with high confidence, despite this being a relatively recent evolutionary event. In contrast, the conformational ensembles sampled of the more diverged RNR-like protein family seldom overlap (fig. 8). Taken together, these results indicate that our MD-based statistical support method is well suited for deeply diverged proteins, and that application to more closely related proteins may produce less robust results, as expected.

### Assessing Statistical Support for the Structural Phylogeny of the Ferritin-Like Superfamily

**Fig. 9.**
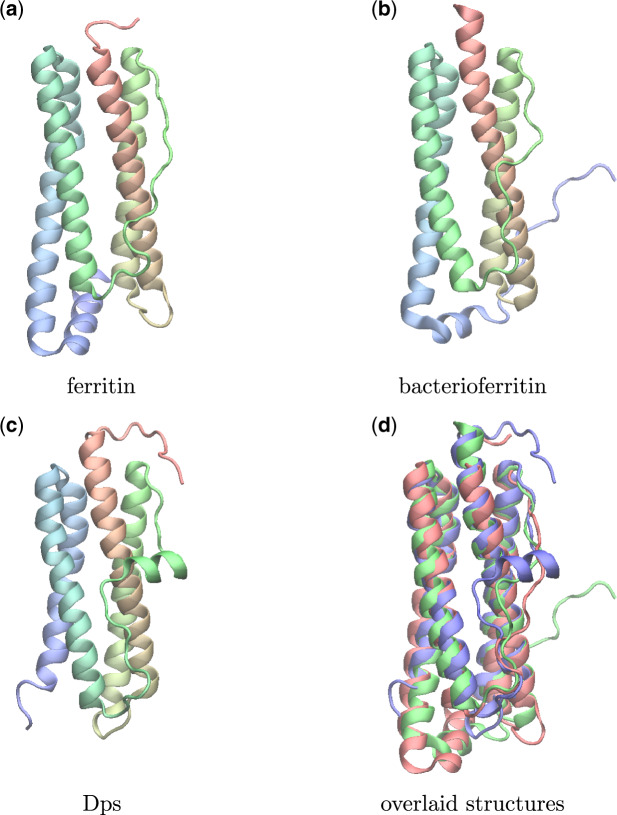
The conserved structural core of proteins in the ferritin-like superfamily comprises a four-helix bundle that coordinates a pair of metal ions. The helices are arranged in a characteristic up-down–down-up topology. Shown here are representative structures from the ferritin (2za7A), bacterioferritin (1nfvA), and Dps (1o9rA) groups colored from (red) N-terminus to (blue) C-terminus (*a*) ferritin, (*b*) bacterioferritin, (*c*) Dps, (*d*) overlaid (ferritin, red; bacterioferritin, green; Dps, blue).

We next applied our MD-based statistical support metric to the much larger ferritin-like superfamily, the structural phylogeny of which was reported previously (Lundin et al. 2012). In addition to the iron-storing ferritins, this superfamily also spans methane mono-oxygenases, the small subunit of RNR R2, rubrerythrins, bacterioferritins, Dps (DNA binding protein from starved cells that protect against oxidative DNA damage), and Dps-like proteins. Across the superfamily, there is very low sequence similarity and substantial differences in quaternary structure and function, but despite this, the ferritins possess a conserved structural core (fig. 9) (Lundin et al. 2012).

**Fig. 10.**
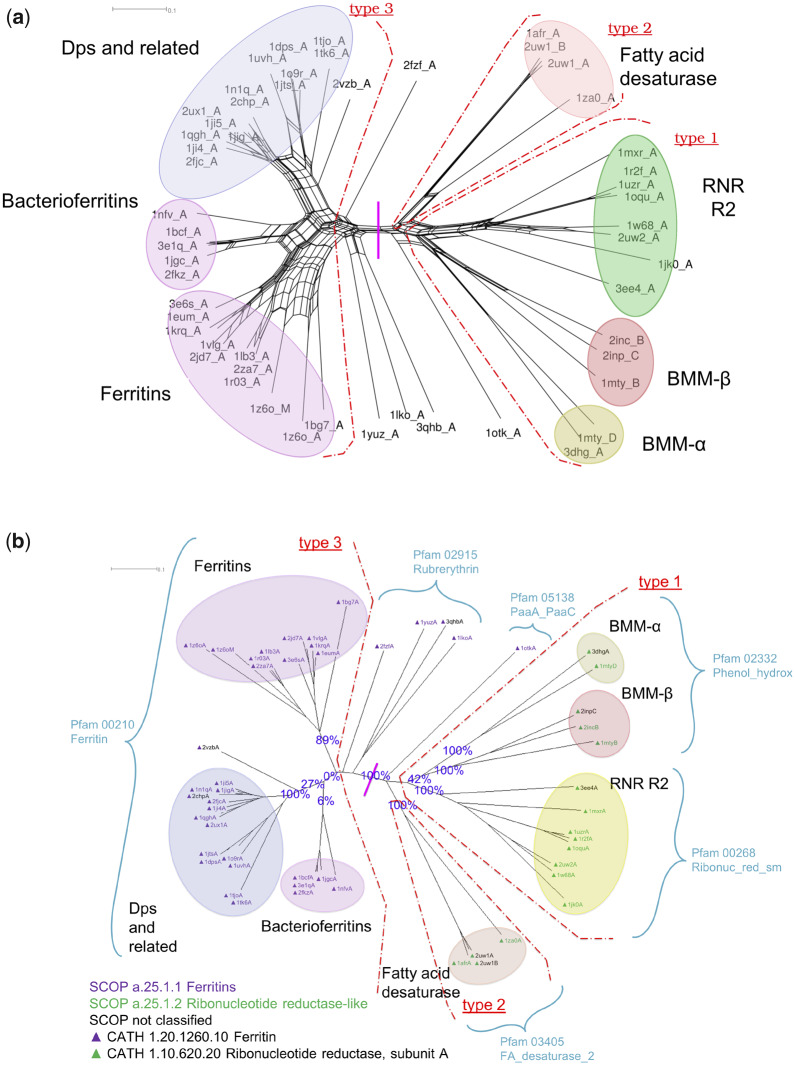
Structure-based phylogenetics of the ferritin-like superfamily. The color-coded ellipses are consistent with the previous study (Lundin et al. 2012) and labeled with annotations provided by the PDB (wwPDB Consortium 2008). The scale bars represent distance as quantified by the inverse *Q*_score_. (*a*) NeighborNet network of the ferritin-like superfamily built from the structures as obtained from the PDB (wwPDB Consortium 2008). The red dot-dashed arcs separate the structures with three different dimerization types whose separate classification was used to assess the quality of the phylogenetic tree by Lundin et al. (2012). The vertical pink line marks the broad split between the two SCOP families, ferritins (a.25.1.1, left) and ribonucleotide reductase-like (a.25.1.2, right). (*b*) Structural phylogeny of the ferritin-like superfamily with statistical support from the structural bootstrap method. The bifurcating tree was built using the structures from which the simulations were initiated, with statistical support generated using MD simulations. Support values obtained from 100 samples of alternative conformations for each protein structure from the repertoire of 10,000 conformations generated during the production phase of the MD simulation are shown for key splits. SCOP and CATH classifications are shown by the color of the node labels and of the associated triangle, respectively, as per the embedded key. Pfam classifications are indicated by arcs.

To assess our method, we derived a core set of protein structures from the ferritin-like superfamily, ran MD simulations for each structure in our data set, and then calculated support for each node in a BioNJ tree derived from the structures deposited in the PDB (fig. 10).

A key conclusion drawn from the previous structural phylogeny of the ferritin-like superfamily (Lundin et al. 2012) was that ferritins, bacterioferritins, and Dps can be grouped together, and that these were distinct from the Fads, RNR R2s, and BMMs, in keeping with their split into two different SCOP families (ferritin [a.25.1.1] and RNR-like [a.25.1.2]) and two different CATH homology groupings (ferritin [1.20.1260.10] and RNR, subunit A [1.10.620.20]). We find 100% support for this split.

The proteins in the SCOP Ferritin family (CATH Ferritin homology) are also grouped together by Pfam (00210 Ferritin). Within the SCOP Ferritin family, our tree shows strong support for the “Ferritins” (89%) and “DPS and related” (100%, if 2vzbA is treated as an outlier) groupings identified previously (Lundin et al. 2012). However, we see minimal support (6%) for the “Bacterioferritins” forming a group, and for these and the “DPS and related” grouping separately from the “Ferritins” (0%). These weak support values are consistent with the high degree of reticulation in this region of the phylogenetic network (fig. 10*a*), and the grouping of all three protein subfamilies within a single “Ferritin” Pfam (002120) (fig. 10*b*).

The SCOP RNR-like family (CATH RNR, subunit A homology) is spread across three different Pfam families (02332 Phenol_hydrox, 00268 Ribonuc_red_sm, and 03405 FA_desaturase_2) (fig. 10*b*). We observe strong support for the monophyly of the fatty acid desaturases (100%) and the RNR R2s (100%), but only moderate support for the monophyly of the BMMs (42%). However, within the BMMs, our tree shows strong support (100%) for their separation into the BMM-α and BMM-β subgroups. This is in keeping with the known duplication of BMMs into BMM-α and BMM-β forms, which was also observed by Lundin et al. (2012). In general, our high support values within the SCOP RNR-like family are in keeping with the relatively low level of reticulation in this region of the phylogenetic network.

### Statistical Support is Robust to Conformational Sampling Method

To test whether our results are influenced by the type of conformational sampling that occurs during MD simulations, we also carried out MC sampling of the conformations accessible to each protein, and built a tree in the same way as for the MD results (supplementary fig. S38, Supplementary Material online).

The statistics that we obtain using MC conformational sampling are somewhat similar to those obtained using MD conformational sampling. We again see strong support (96%) for the split between the SCOP ferritin (a.25.1.1) and RNR-like (a.25.1.2) families (CATH ferritin [1.20.1260.10] and RNR, subunit A [1.10.620.20] homology groupings). The main groupings within the SCOP RNR-like family have equivalent levels of support (100%) under both MD and MC sampling. Likewise, the main groupings within the SCOP ferritin family are consistent across both MD and MC. However, with MC, support for the Ferritins is reduced (50% cf. 89%), as is support for the “Dps and related” group (52% without 2vzbA). The support for the “Bacterioferritins” (14%), and for these and “DPS and related” grouping to the exclusion of the “Ferritins” (2%) is low, consistent with the low support values obtained with MD sampling. The support values for the location of 2vzbA relative to the “Dps and related” group vary greatly between the MD and MC sampling. The position of 2vzbA (which is not classified by SCOP) thus remains uncertain.

Overall, we found that MC yields similar support values to MD sampling. We found, however, that MC required more computational resources to reach an equivalent level of conformational sampling to the MD simulations.

### Resolution of SCOP and CATH Polytomies

Our reduced ferritin-like protein structure data set spans one SCOP superfamily, ferritin-like (a.25.1), comprising two manually curated protein families, ferritin (a.25.1.1) and RNR-like (a.25.1.2) (supplementary table S5, Supplementary Material online). Similarly, it spans one CATH topology group that is split into two homology groups, Ferritin (1.20.1260.10) and RNR, subunit A (1.10.620.20). In Pfam, it spans six families, ferritins (PF00210), Ribonuc_red_sm (PF00268), Rubrerythrin (PF02915), Phenol_Hydrox (PF02332), Fatty acid desaturase (PF03405), and PaaA_PaaC (PF05138), which all belong to a single Pfam clan, ferritin (CL0044).

All three classification systems have just one Ferritin family, which we reproduce at a high level. Our results suggest that this group could be further split into three subgroups, separating out “Dps and related” and “Bacterioferritins” from the remainder of the Ferritins.

In contrast, although SCOP and CATH have a single-overarching RNR-like family, these proteins are classified into three distinct families by Pfam, Phenol_Hydrox (PF02332), Ribonuc_red_sm (PF00268), and Fatty acid desaturase (PF03405). We find consistently high support for this more detailed sequence-based classification, as well as the further separation of the BMMs into BMMα and BMMβ.

There are several proteins that lie outside of the major groupings in our networks, all of which are classified by CATH as Ferritins, and most of which are also classified by SCOP as ferritins. We find strong support for one of these, 1otkA, grouping with the RNRs rather than the ferritins. 1otkA was also found to lie close to BMMα (Lundin et al. 2012), a result which we also recover (fig. 10*b*). Pfam classifies these proteins into two groups, rubrerythrin, and PaaA_PaaC, with 1otkA the only member of PaaA_PaaC. As well as sequence differences, the Pfam groupings correspond to quite different dimer topologies to the three major types indicated in figure 10, and particularly different to the simple type 3 topology of the ferritins. We therefore suggest that these outliers might be better categorized separately to the major ferritin groupings in SCOP and CATH.

## Conclusions

We have developed a novel method for generating statistical support to distance-based, protein structural phylogenies. This procedure requires a metric for quantifying protein structural similarity that fulfills three key criteria, and a means of generating alternative conformations for each protein so that multiple trial trees can be constructed, analogous to the bootstrap method used for sequence data.

Protein structural similarity was determined using the *Q*_score_ (Krissinel and Henrick 2004), which can be divided into two parts, *Q*_length_ (eq. 2) and *Q*_shape_ (eq. 3), which account for the contributions from differences in the number of amino acids and morphometric differences. The influence of each part on the overall *Q*_score_ was evaluated in a controlled manner. We find that if the size difference between the compared protein structures varies by >10%, the size contribution to *Q*_score_ dominates. We note, however, that our test involved unidirectional truncation of protein structures, with comparison back to untruncated structures, which we expect to be quite a stringent test. The *Q*_score_ value can also be influenced by variation in the protein structure of the degree expected to occur during an MD simulation, with the important implication that *Q*_score_-based structural phylogenetic methods are unlikely to be suitable for investigation of recently diverged proteins. In this case, sequence-based comparisons are both appropriate and more suitable.

We used MD simulations to generate alternative conformations for each protein structure. We show that in the case of the RNR-like family, which is sufficiently diverged to be suitable for structural phylogenetic analysis, the extent of conformational sampling during a short 100-ns simulation is enough to generate conformations sufficiently different for us to observe perturbations in the structural distance data while retaining a folded state, which is required for meaningful structural comparisons.

As expected, a test of the MD-based bootstrap method resulted in weak support values for the phylogeny of the recently diverged proteins from the globin family, with stronger support values for the phylogeny of the more highly diverged RNR-like family. This provided further confirmation that structure-based phylogenetic inference is most suitable for highly diverged proteins for which sequence-based methods may struggle.

We used our MD-based bootstrap method to add statistical support to the structural phylogeny of the ferritin-like protein superfamily. The qualitative assessment of the phylogenetic relationships made by Lundin et al. (2012) based on the topology of the proteins on the reticulated network was supported by our results, with separations that have a tree-like structure in the reticulated network having strong statistical support, and those where the network departs from tree-likeness having weaker support. We found similar support values when we used MC rather than MD to sample alternative conformations for each protein.

Our MD-based bootstrap method may augment the deep evolutionary classifications of protein structure in databases like SCOP, CATH, and Pfam. As a proof-of-principle test of our method, we successfully recovered support the major relationships across the ferritin-like protein superfamily in SCOP and the analogous Ferritin homology grouping in CATH. We found similarly strong support for the RNR-like superfamily in SCOP and analogous RNR subunit A homology grouping in CATH, plus support for the finer classification of the RNR-like superfamily by Pfam. In addition, we observed strong support for the subdivision of the BMMs into BMMα and BMMβ, a level of detail lacking from all three databases.

Structural phylogenetics provides a means of probing deep evolutionary relationships where sequence similarity is too low to confidently apply sequence-based methods of phylogenetic analysis. Here, we have implemented and validated a method for providing statistical support values for structural phylogeny using MD and MC simulations to sample alternative conformations for each protein, allowing the robustness of the inferred relationships to be assessed. Our method may augment the hierarchical classification of structures within structural databases, resolving phylogenetic relationships where sequence data cannot.

## Materials and Methods

### Selection and Processing of Protein Structures

#### Structures of Representative Size to Test *Q*_length_

The PDB format files of all 102,540 structures in the PDB (as of July 18, 2019) were downloaded from the PDB (wwPDB Consortium 2018). Non-protein elements were removed, and the remaining proteins separated into chains, resulting in 290,306 structures. The length of each of these structures in terms of the number of amino acids was then determined. Small proteins (<40 amino acids) were removed, as these did not represent complete domains. Similarly, protein structures with >350 amino acids were assumed to be multidomain proteins and hence were excluded. This resulted in 150,000 single-chain, single-domain protein structures, which constitute the central and most densely populated part of the length distribution (supplementary fig. S1, Supplementary Material online). Using the *K*-means clustering algorithm (Forgy 1965; Lloyd 1982), three centroids of the length distribution were determined using Scipy (0.13.3) (Virtanen et al. 2020) with Python2.7. This produced centroids at lengths of 125, 184, and 234 amino acids, respectively, which served as estimates of common protein lengths.

The SCOP (Andreeva et al. 2019) annotations were automatically searched for families having proteins with lengths distributed around these common length values. This identified the globin, trypsin-like serine protease, and aldo-keto reductase (NADP) families. From each family, 20 structures (for convenience only) were selected, which are listed in supplementary table S1, Supplementary Material online, along with their respective lengths. Each structure was decomposed into ten fractions, with a given fraction comprising the first *n*% * *N* amino acids of the protein, starting from the most N-terminal residue present in the structure, where {n∈10,20,…,100%} and *N* is the number of residues in the structure (supplementary fig. S2, Supplementary Material online). Each set of fractional structures was combined with the set of complete structures and used to generate a phylogenetic tree.

#### Structures to Test *Q*_shape_

The same 53 protein structures used to build a phylogeny of the ferritin-like superfamily (see below) were used to explore the effect of shape on *Q*_score_.

#### Structures to Test MD-Based Bootstrap Method

The PDB IDs of the eight RNR-like and eight globin structures used to test the MD-based bootstrap method are listed in supplementary table S4, Supplementary Material online.

#### Ferritin-Like Superfamily

The structures of 83 proteins from the ferritin-like SCOP superfamily examined in the previous structural phylogenetic study by Lundin et al. (2012) were obtained from the PDB (wwPDB Consortium 2018). These are listed along with their SCOP, CATH, and Pfam classifications in supplementary tables S2 and S5, Supplementary Material online. Not all of the 83 protein structures used in the previous study were included in this analysis. Protein structures belonging to families uncharacterized by SCOP, or having fewer than three members, were removed, with the latter criterion ensuring that only families for which the internal hierarchy can be meaningfully resolved were included. Furthermore, some structures were not able to be simulated due to missing residues (e.g., 1jk0B) or problems with the structural geometry (e.g., 1mhyB and 1mhyD). Despite extensive energy minimization, some of the geometry-related problems could not be corrected. In total, 53 protein structures that have clean structural geometries, are characterized by at least two of the SCOP, CATH, and Pfam databases, and are part of groups for which (Lundin et al. 2012) drew important inferences were retained. The excluded structures are marked with “*” in supplementary table S2, Supplementary Material online.

### Simulation Procedures

#### Simulations

MD simulations were carried out using the GROMACS (Abraham et al. 2015) program along with the CHARMM36 (Best et al. 2012) force field and the TIP3P water model (Jorgensen et al. 1983). All simulations were conducted in an NpT ensemble to mimic physiological conditions, at a pressure of 1 atm. The Lennard–Jones potential was switched to zero between 10 and 12 Å and a 12 Å cut-off distance was used for calculating the electrostatic interactions. Electrostatic interactions outside the cut-off were computed using particle mesh Ewald (Darden et al. 1993) summation. Temperature was maintained at 310 K using the velocity rescale modified Berendsen thermostat with a coupling constant τ_t_ of 0.1 ps and pressure with a Berendsen barostat with a coupling constant τ_p_ of 0.5 ps and an isothermal compressibility of $4.5\times 10^{-5}$ (kJmol^−1^nm^−3^)^−1^. The lengths of covalent bonds involving hydrogen atoms were constrained using LINCS (Hess et al. 1997) to allow for an integration time step of 2 fs.

Each structure was energy minimized using the steepest-descent algorithm for 5,000 steps or until the energy changed by <2 kJmol^−1^. A minimum cubic box was created around the protein, the boundaries extended by 15 Å in each direction, the box filled with solvent molecules, and energy minimized again for 5,000 steps. Excess charge was neutralized (if present) through the addition of Na^+^ and/or Cl^−^ counter ions by randomly selecting a water molecule and substituting it with an ion. The system was minimized again for 5,000 steps to remove any clashes. The system was simulated for 10 ps at 50 K then annealed from 50 to 310 K over 200 ps and equilibrated at 310 K for a further 40 ps. Finally, the system was simulated for 100 ns, with conformations recorded every 10 ps to give 10,000 conformations in total.

MC simulations were conducted using Phaistos (Boomsma et al. 2013) using the OPLS (Jorgensen 2002) force field via the Phaistos opls-mc-dynamics mode. OPLS is an established biomolecule force field similar to the CHARMM36 force field used for the MD simulations, thus the major difference between these two data sets is the sampling method. Conformational sampling was carried out using pivot, semilocal, and local backbone moves in internal coordinate space. Five independent replica simulations comprising 5,000,000 steps each were carried out for each protein structure, initiated from the PDB coordinates. Sampled conformations were recorded every 10,000 steps, resulting in a pool of 2,500 conformations for each protein.

#### Analysis

The coordinate trajectory for each system was analyzed with GROMACS (Abraham et al. 2015) and VMD (Humphrey et al. 1996) using standard procedures and in-house Tcl scripts, which are available at https://github.com/allison-group/structural-phylogenetics-bootstrap. RMSD calculations were for the Cα atoms only, to match the RMSD calculations carried out by Superpose (Krissinel and Henrick 2004). Conformational clustering was carried out using the “gromos” method (Daura et al. 1999), as implemented in GROMACS.

### Phylogenetic Procedures

#### Generation of Phylogenetic Trees

Pairs of protein structures were compared using Superpose (Krissinel and Henrick 2004). Due to the nature of the algorithm, comparisons are order specific, that is, A ≅ B ≠ B ≅ A. Therefore, both pairwise comparisons were performed and the *Q*_score_ values were averaged to attain a final *Q*_score_ value *q* for the comparison between structure A and B. The distance between the pair of structures was then calculated as d=1−q. A matrix was populated with the pairwise distances and a neighbor-joining (NJ) tree generated using the NJ algorithm (Saitou and Nei 1987) as implemented by the Phylo package (Talevich et al. 2012) in Biopython (Cock et al. 2009). Trees were visualized using Figtree (Rambaut 2007) and Dendroscope (Huson et al. 2007; Huson and Scornavacca 2012).

#### Quantitative Comparison of Phylogenetic Trees

In evaluating the effect of *Q*_length_, the phylogenetic tree comparison program “treecompare” as made available by DendroPy (Sukumaran and Holder 2010), a python library for phylogenetic computing, was used to calculate the Euclidean distance to quantify the difference between the fractional trees and the true tree.

In all other cases, the relationships between protein structures in the replicate trees were compared with those in the reference tree, *T*_0_, using the phylogenetic tree summarization program SumTrees via DendroPy (Sukumaran and Holder 2010). The recovered relationships were expressed as a percentage of the total number of trials on the nodes in *T*_0_.

#### MD-Based Bootstrap Method

The code used to carry out our MD-based bootstrap method for structural phylogenetics is available from https://github.com/allison-group/structural-phylogenetics-bootstrap. It requires Python v2.7, VMD (Humphrey et al. 1996) (v1.9.2 or later), the Phylo (Talevich et al. 2012) package from Biopython (Cock et al. 2009), and the DendroPy (Sukumaran and Holder 2010) package. Calls to VMD (Humphrey et al. 1996) programs were made via the bash shell.

## Supplementary Material

Supplementary data are available at *Molecular Biology and Evolution* online.

## Supplementary Material

msaa100_Supplementary_Data
