# Supplementary material for: Structural Phylogenetics with Confidence
Source: Mol Biol Evol. 2020 Apr 17;37(9):2711–26. doi: 10.1093/molbev/msaa100 (PMC7475046; doi:10.1093/molbev/msaa100)
Supplement: msaa100_Supplementary_Data [file msaa100_supplementary_data.zip › msaa100-Suppl_Data/StructuralPhylogeny_MBE_SI_FINAL.pdf]

Supplementary Material for: Structural phylogenetics with confidence

Ashar J Malik[1,2], Anthony M Poole*[3,4,5], and Jane R Allison†[3,4,5,6]

[1]Centre for Theoretical Chemistry and Physics, School of Natural and Computational Sciences, Massey University Auckland, Private Bag 102904, 0632 Auckland, New Zealand
[2]Bioinformatics Institute, Agency for Science, Technology and Research, 138671 Singapore
[3]Bioinformatics Institute, School of Biological Sciences, University of Auckland, Private Bag 92019, Auckland 1142, New Zealand
[4]Digital Life Institute, University of Auckland, Private Bag 92019, Auckland 1142, New Zealand
[5]Biomolecular Interaction Centre, University of Canterbury, Private Bag 4800, Christchurch 8140, New Zealand
[6]Maurice Wilkins Centre for Molecular Biodiscovery, University of Auckland, Private Bag 92019, Auckland, New Zealand

March 10, 2020

Supplementary Material

Table S 1: PDB accession codes, chain identifiers and lengths of the proteins from the three protein families, globins, trypsin-like serine proteases and aldo-keto reductases (NADP) used to test the contribution of Q_{length} to Q_{score}.

**Globins**			**Trypsin-like serine proteases**			**Aldo-keto reductases (NADP)**		
**PDB**	**Chain**	**Length**	**PDB**	**Chain**	**Length**	**PDB**	**Chain**	**Length**
1dxv	B	146	3hgp	A	240	3m4h	A	316
4mqi	A	140	4z6a	H	249	1mi3	A	322
3sdh	A	146	1a0l	A	244	2hdj	A	323
1uc3	A	149	4crg	A	238	2hej	A	323
5eys	A	148	1t8o	C	245	4xzm	X	316
3cy5	A	141	2wph	S	235	4gac	A	324
3d1a	B	145	3p8g	A	241	1ah0	A	316
2h8f	A	143	1l4z	A	248	4mhb	A	297
1hbr	B	146	5lhr	A	247	1afs	A	323
2h8f	B	146	4dgj	A	235	1q13	A	323
2d5x	B	146	1fon	A	240	1og6	A	298
2d5x	A	141	1m9u	A	241	3o3r	A	316
3tm9	A	146	1ao5	A	237	fvl	A	324
3gqp	B	145	4nfe	A	237	4r9o	A	301
3gqp	A	141	1pfx	C	235	1vp5	A	298
1i3d	A	146	1sgf	G	237	1gve	A	327
1myt	A	146	4bnr	A	237	1c9w	A	315
1a4f	A	141	5gvt	A	250	1mzr	A	296
4f4o	B	146	1sgf	A	240	3ln3	A	324
1qpw	A	141	3w94	A	235	1qwk	A	317

*a.poole@auckland.ac.nz
†j.allison@auckland.ac.nz

Table S 2: PDB accession codes and chain, SCOP, CATH and Pfam identifiers of protein structures used previously by Lundin et al. (Lundin et al., 2012) to build a structural phylogeny of the ferritin-like family. Protein structures unclassified by SCOP are left blank. “*” indicates structures that were excluded from our analysis due a) belonging to families uncharacterised by SCOP; (b) having fewer than three members; (c) being unsuitable for MD simulation due to missing residues (e.g. 1jk0_B) or problems with the structural geometry (e.g. 1mhy_B and 1mhy_D). Colours are for visual guidance only.

PDB	Chain	SCOP	CATH	Pfam	PDB	Chain	SCOP	CATH	Pfam
1bcf	A	a.25.1.1	1.20.1260.10	PF00210	1uzr	A	a.25.1.2	1.10.620.20	PF00268
1bg7	A	a.25.1.1	1.20.1260.10	PF00210	1w68	A	a.25.1.2	1.10.620.20	PF00268
1dps	A	a.25.1.1	1.20.1260.10	PF00210	*2rcc	A		1.10.620.20	PF00268
1eum	A	a.25.1.1	1.20.1260.10	PF00210	2uw2	A	a.25.1.2	1.10.620.20	PF00268
1jgc	A	a.25.1.1	1.20.1260.10	PF00210	*3dhz	A	a.25.1.2	1.10.620.20	PF00268
1ji4	A	a.25.1.1	1.20.1260.10	PF00210	3ee4	A		1.10.620.20	PF00268
1ji5	A	a.25.1.1	1.20.1260.10	PF00210	*1z3a	A	c.97.1.2	3.41.140.10	PF00383
1jig	A	a.25.1.1	1.20.1260.10	PF00210	*1mhy	B	a.25.1.2	1.10.620.20	PF02332
1jts	A	a.25.1.1	1.20.1260.10	PF00210	*1mhy	D	a.25.1.2	1.10.620.20	PF02332
1krq	A	a.25.1.1	1.20.1260.10	PF00210	1mty	B	a.25.1.2	1.10.620.20	PF02332
1lb3	A	a.25.1.1	1.20.1260.10	PF00210	1mty	D	a.25.1.2	1.10.620.20	PF02332
1n1q	A	a.25.1.1	1.20.1260.10	PF00210	*1xvb	A	a.25.1.2	1.10.620.20	PF02332
1nfv	A	a.25.1.1	1.20.1260.10	PF00210	*1xvb	C	a.25.1.2	1.10.620.20	PF02332
1o9r	A	a.25.1.1	1.20.1260.10	PF00210	*2inc	A	a.25.1.2	1.10.620.20	PF02332
1qgh	A	a.25.1.1	1.20.1260.10	PF00210	2inc	B	a.25.1.2	1.10.620.20	PF02332
1r03	A	a.25.1.1	1.20.1260.10	PF00210	*2inp	A		1.10.620.20	PF02332
*1rci	A	a.25.1.1	1.20.1260.10	PF00210	2inp	C		1.10.620.20	PF02332
*1s3q	A	a.25.1.1	1.20.1260.10	PF00210	3dhg	A	a.25.1.2	1.10.620.20	PF02332
1tjo	A	a.25.1.1	1.20.1260.10	PF00210	*3dhg	B	a.25.1.2	1.10.620.20	PF02332
1tk6	A	a.25.1.1	1.20.1260.10	PF00210	1lko	A	a.25.1.1	1.20.1260.10	PF02915
1uvh	A	a.25.1.1	1.20.1260.10	PF00210	*1vjx	A	a.25.1.1	1.20.1260.10	PF02915
1vlg	A	a.25.1.1	1.20.1260.10	PF00210	1yuz	A	a.25.1.1	1.20.1260.10	PF02915
1z6o	A	a.25.1.1	1.20.1260.10	PF00210	2fzf	A	a.25.1.1	1.20.1260.10	PF02915
1z6o	M	a.25.1.1	1.20.1260.10	PF00210	*2oh3	A	a.25.1.8	1.20.1260.10	PF02915
2chp	A	a.25.1.0	1.20.1260.10	PF00210	3qhb	A		1.20.1260.10	PF02915
*2clb	A		1.20.1260.10	PF00210	1afr	A	a.25.1.2	1.10.620.20	PF03405
2fjc	A	a.25.1.1	1.20.1260.10	PF00210	*1oqb	A	a.25.1.2	1.10.620.20	PF03405
2fkz	A	a.25.1.1	1.20.1260.10	PF00210	1za0	A	a.25.1.2	1.10.620.20	PF03405
2jd7	A	a.25.1.0	1.20.1260.10	PF00210	2uw1	A	a.25.1.2	1.10.620.20	PF03405
*2qqy	A		1.20.1260.10	PF00210	2uw1	B	a.25.1.2	1.10.620.20	PF03405
2ux1	A	a.25.1.1	1.20.1260.10	PF00210	*2qf9	A		1.20.1260.10	PF03713
2vzb	A		1.20.1260.10	PF00210	*1o9i	A	a.25.1.3	1.20.1260.10	PF05067
2za7	A	a.25.1.1	1.20.1260.10	PF00210	*2cwl	A	a.25.1.3	1.20.1260.130	PF05067
3e1q	A	a.25.1.1	1.20.1260.10	PF00210	1otk	A	a.25.1.2	1.20.1260.10	PF05138
3e6s	A	a.25.1.1	1.20.1260.10	PF00210	*2gs4	A	a.25.1.4	1.20.1260.10	PF05974
*3fvb	A	a.25.1.0	1.20.1260.10	PF00210	*2gyq	A	a.25.1.4	1.20.1260.10	PF05974
1jk0	A	a.25.1.2	1.10.620. 20	PF00268	*2itb	A	a.25.1.7	1.20.1260.10	PF06175
*1jk0	B	a.25.1.2	1.10.620.20	PF00268	*3fse	A		1.20.1260.10	PF09537
1mxr	A	a.25.1.2	1.10.620.20	PF00268	*2oc5	A	a.25.1.6	1.20.1260.10	PF11266
1oqu	A	a.25.1.2	1.10.620.20	PF00268	*3ez0	A		1.20.1260.10	PF13794
1r2f	A	a.25.1.2	1.10.620.20	PF00268	*2ib0	A	a.25.1.9	1.20.1260.10	PF14530
*1syy	A	a.25.1.2	1.10.620.20	PF00268					

Table S 3: Range of raw RMSD values corresponding to each bin, and the number of ferritin-like proteins that sample RMSD values in this range during MD simulation.

RMSD range (nm)	Bin number	Number of proteins
0.5-1.0	1	53
1.0-1.5	2	53
1.5-2.0	3	53
2.0-2.5	4	48
2.5-3.0	5	40
3.0-3.5	6	38
3.5-4.0	7	30
4.0-4.5	8	20
4.5-4.0	9	18
5.0-5.5	10	12

Table S 4: PDB accession codes and chain identifiers of the ribonucleotide reductase-like (RNR-like) and globin structures used to test the MD-based bootstrap method. In the globins column, chains with identifier "A" are α-haemoglobins and those with identifier "B" are β-haemoglobins.

**RNR-like**		**Globins**	
**PDB**	**Chain**	**PDB**	**Chain**
1afr	A	1gcv	A
1mty	B	1gcv	B
1r2f	A	1hv4	A
1uzr	A	1hv4	B
1za0	A	2dn2	A
2inp	C	2dn2	B
2uw1	A	3hrw	A
2uw2	A	3hrw	B

Table S 5: SCOP superfamily (SF) and Family, CATH Homology, and Pfam Clan and Family categorisation of the ferritin-like protein structures used to generate a structural phylogeny with statistical support using the MD-based bootstrap method.

	**SCOP**	
SF	Family	Annotation
a.25.1	a.25.1.1	Ferritin
	a.25.1.2	Ribonucleotide reductase-like
	a.25.1.0	automated matches

	**CATH**	
	Homology	Annotation
	1.20.1260.10	Ferritin
	1.10.620.20	Ribonucleotide reductase, subunit A

	**Pfam**	
Clan	Family	Annotation
CL0044	PF00210	Ferritin
	PF00268	Ribonuc_red_sm
	PF02915	Rubrerythrin
	PF02332	Phenol_Hydrox
	PF05067	Mn_catalase
	PF05138	PaaA_PaaC
	PF03405	Fatty acid desaturase

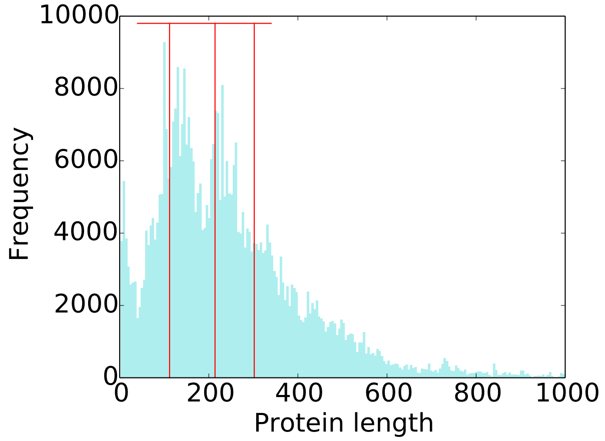

Figure S 1: Distribution of lengths of all 311,634 proteins present in the PDB(wwPDB consortium, 2018) (accession date 10 September 2019) with lengths between 40 and 350 amino acids. The densest portion of the distribution is indicated by the red horizontal bar, and the centroids of each cluster within this region are shown with red vertical lines at protein sizes of 112, 214 and 302 amino acids.

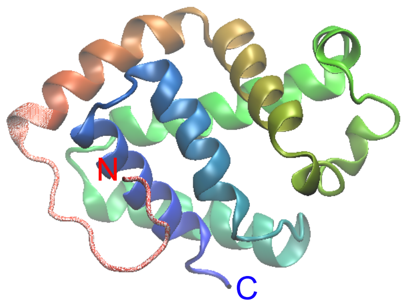

(a)

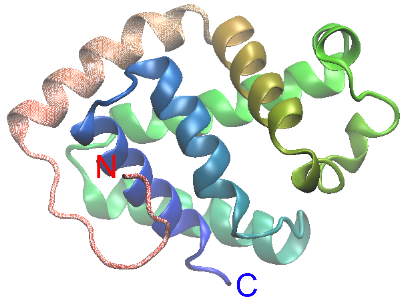

(b)

Figure S 2: Illustration of fractional protein structures for PDB ID 1uc3 (Lamprey haemoglobin, chain A). The complete structure (149 residues) is coloured from red (N-terminus) to blue (C-terminus), and the (a) 10% fractional structure (15 residues) and (b) 20% structure (30 residues) are overlaid as silver hatching.

Globins

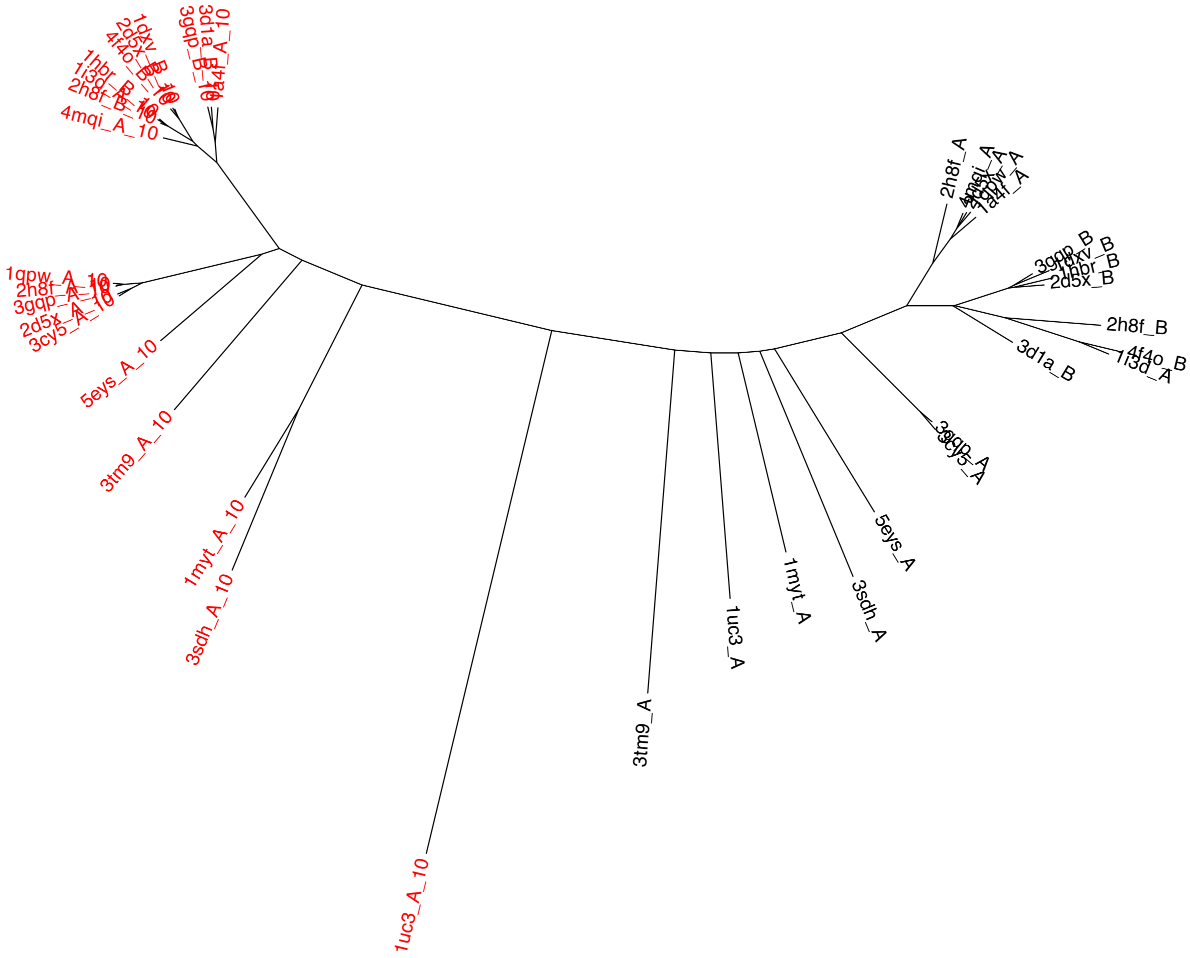

Figure S 3: Phylogenetic tree for proteins from the globin family built using structural datasets comprising a 10% of each structure together with the complete structures. The fractional structures are shown in red and the complete structures in black.

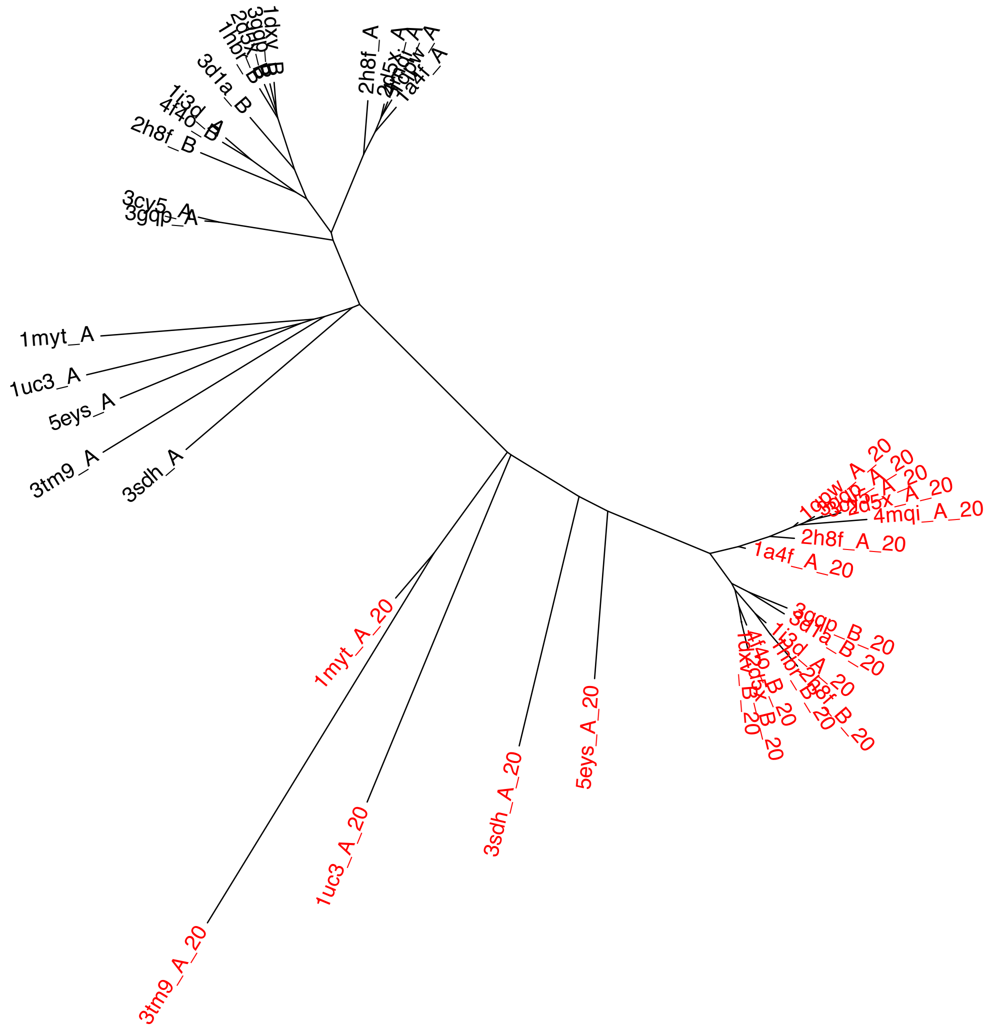

Figure S 4: Phylogenetic tree for proteins from the globin family built using structural datasets comprising a 20% of each structure together with the complete structures. The fractional structures are shown in red and the complete structures in black.

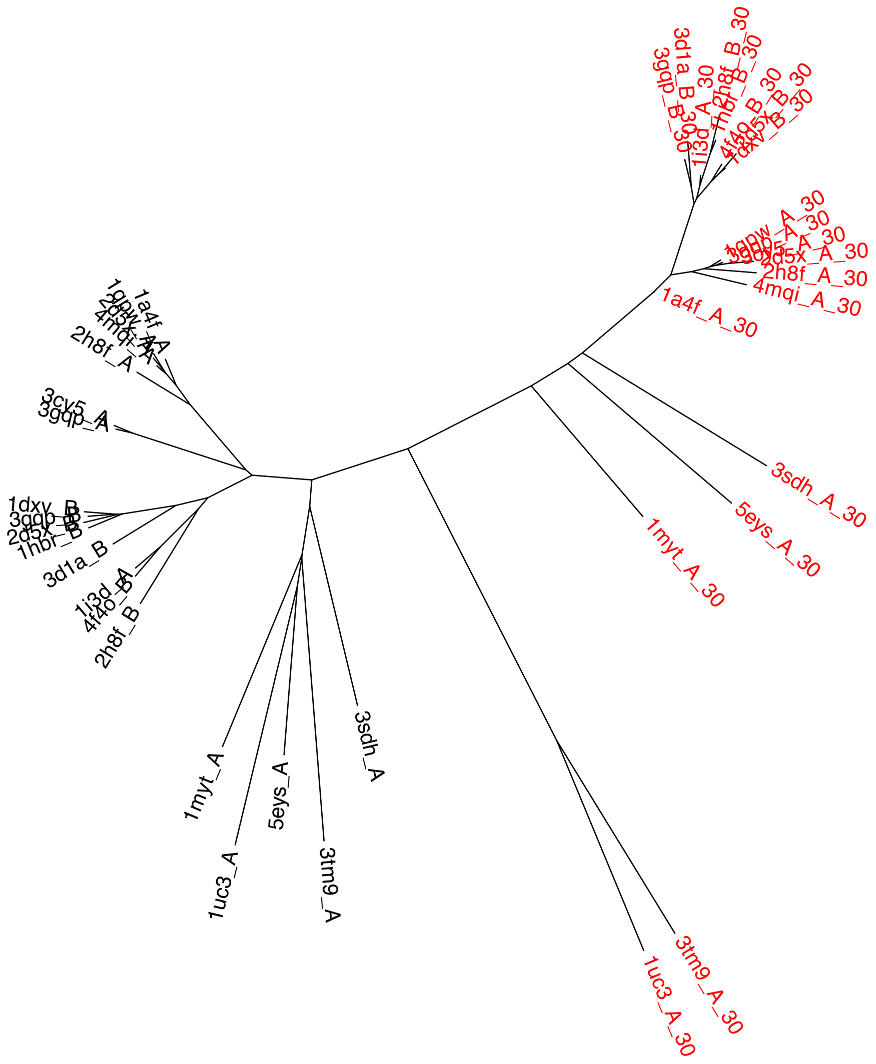

Figure S 5: Phylogenetic tree for proteins from the globin family built using structural datasets comprising a 30% of each structure together with the complete structures. The fractional structures are shown in red and the complete structures in black.

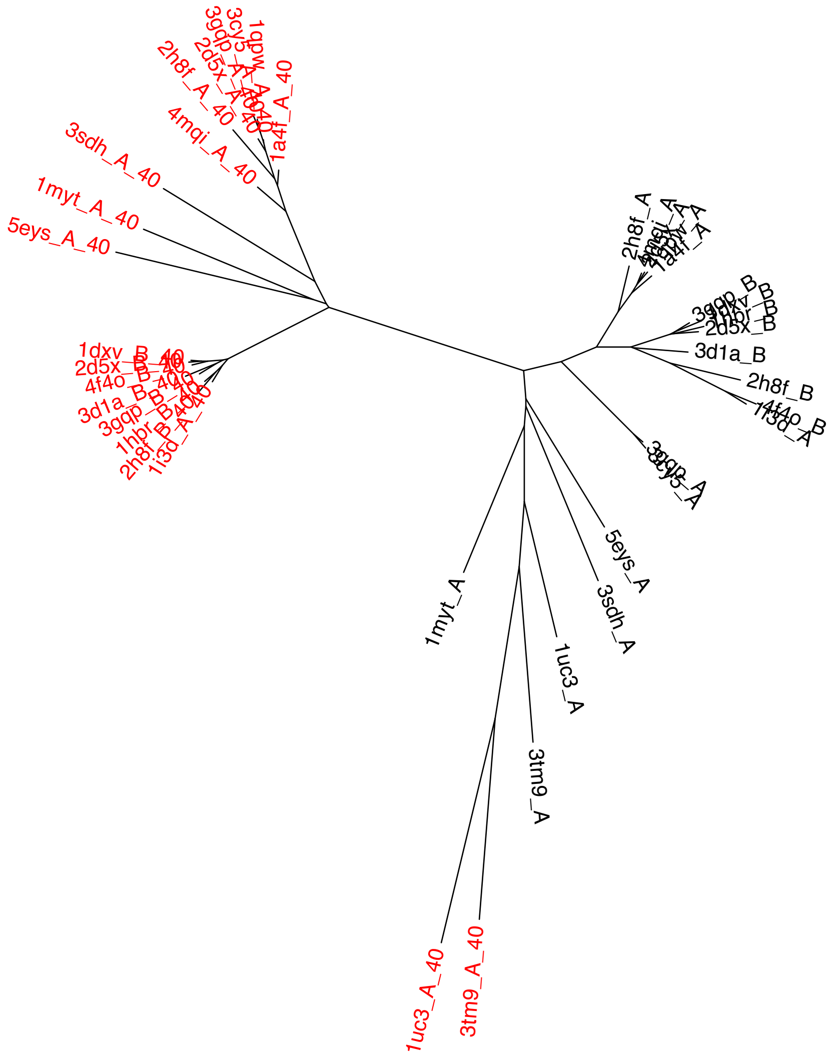

Figure S 6: Phylogenetic tree for proteins from the globin family built using structural datasets comprising a 40% of each structure together with the complete structures. The fractional structures are shown in red and the complete structures in black.

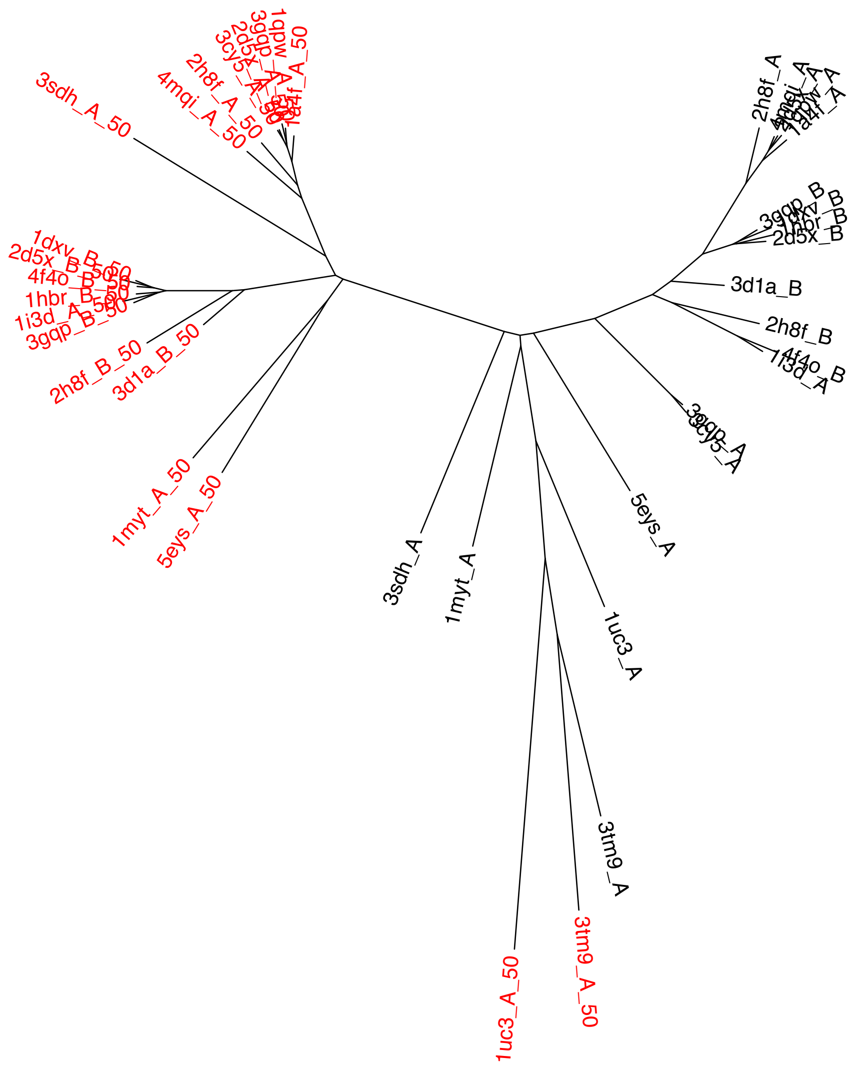

Figure S 7: Phylogenetic tree for proteins from the globin family built using structural datasets comprising a 50% of each structure together with the complete structures. The fractional structures are shown in red and the complete structures in black.

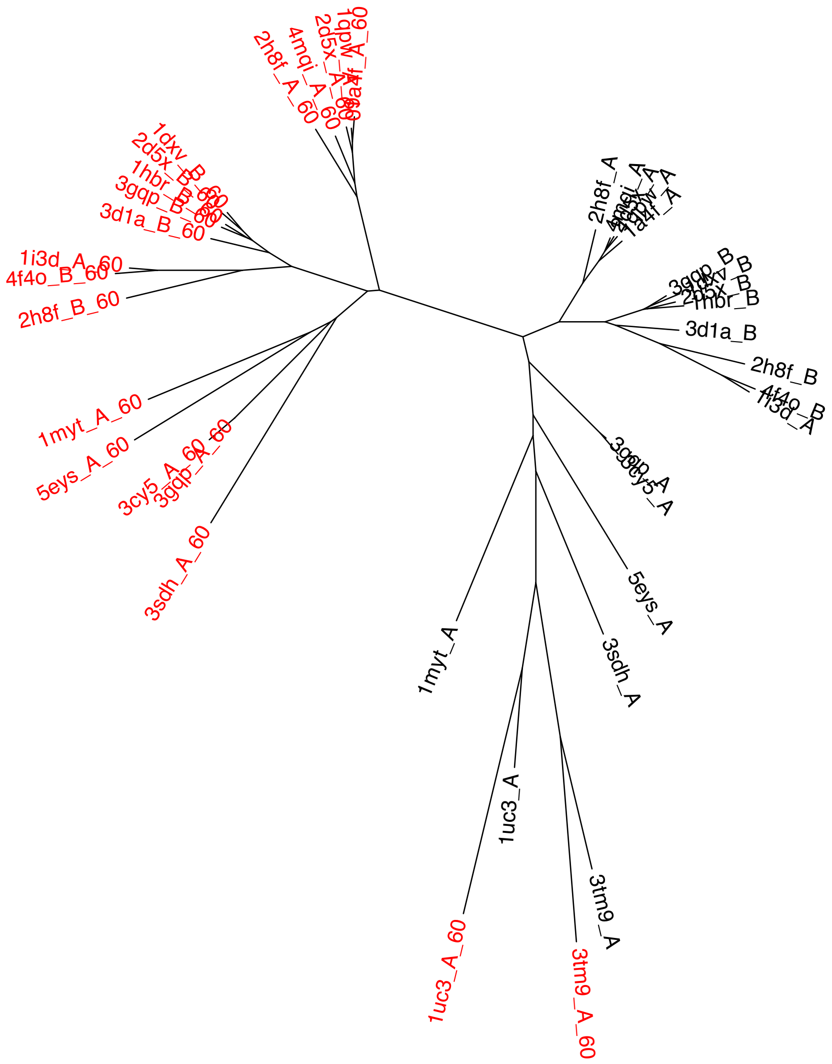

Figure S 8: Phylogenetic tree for proteins from the globin family built using structural datasets comprising a 60% of each structure together with the complete structures. The fractional structures are shown in red and the complete structures in blackk.

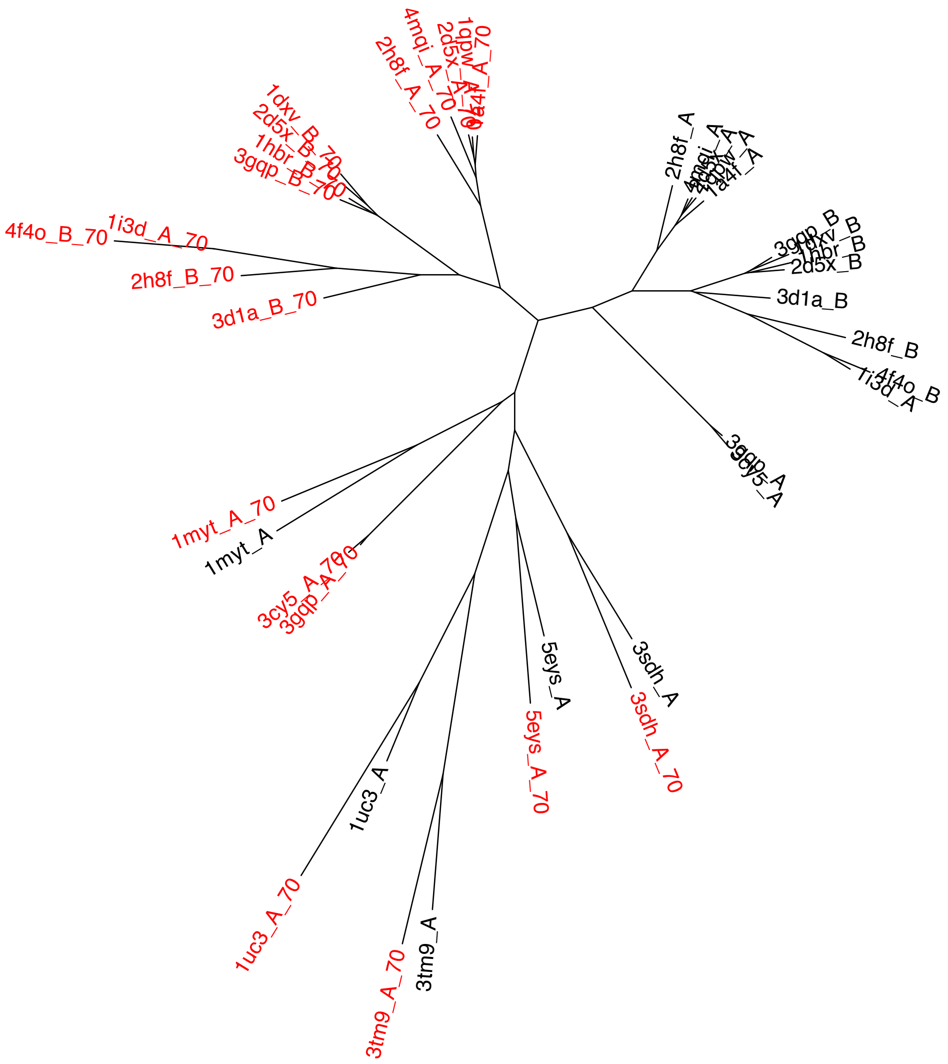

Figure S 9: Phylogenetic tree for proteins from the globin family built using structural datasets comprising a 70% of each structure together with the complete structures. The fractional structures are shown in red and the complete structures in black.

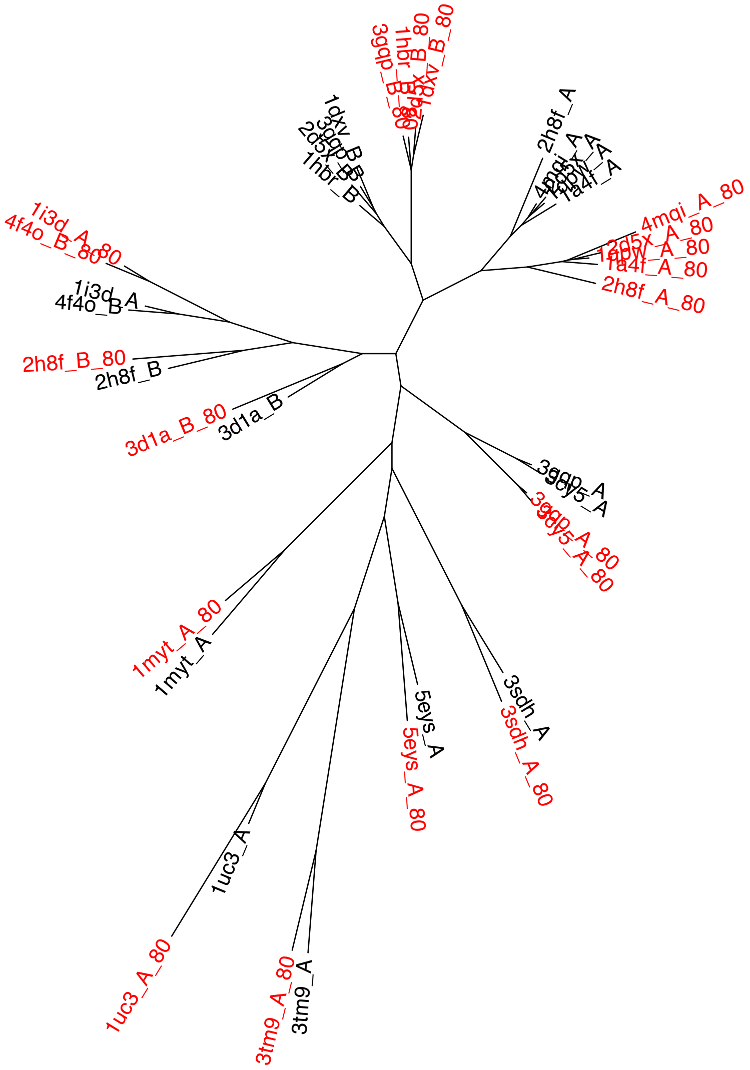

Figure S 10: Phylogenetic tree for proteins from the globin family built using structural datasets comprising a 80% of each structure together with the complete structures. The fractional structures are shown in red and the complete structures in black.

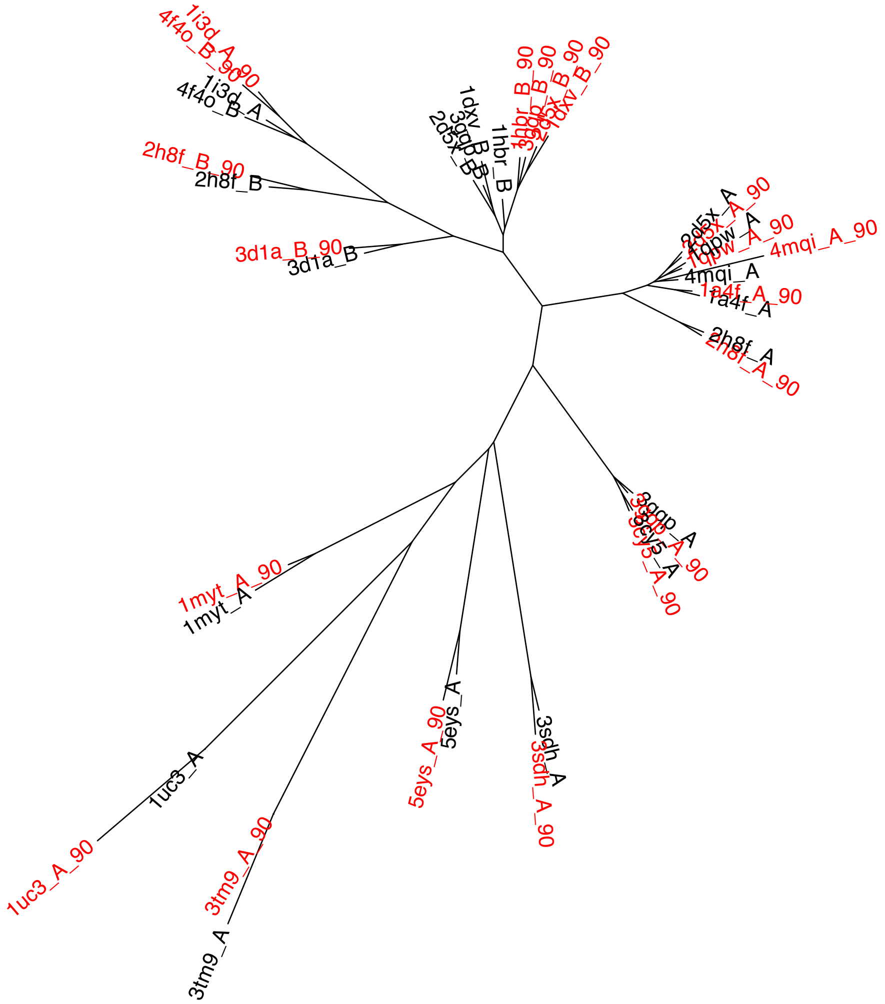

Figure S 11: Phylogenetic tree for proteins from the globin family built using structural datasets comprising a 90% of each structure together with the complete structures. The fractional structures are shown in red and the complete structures in blackk.

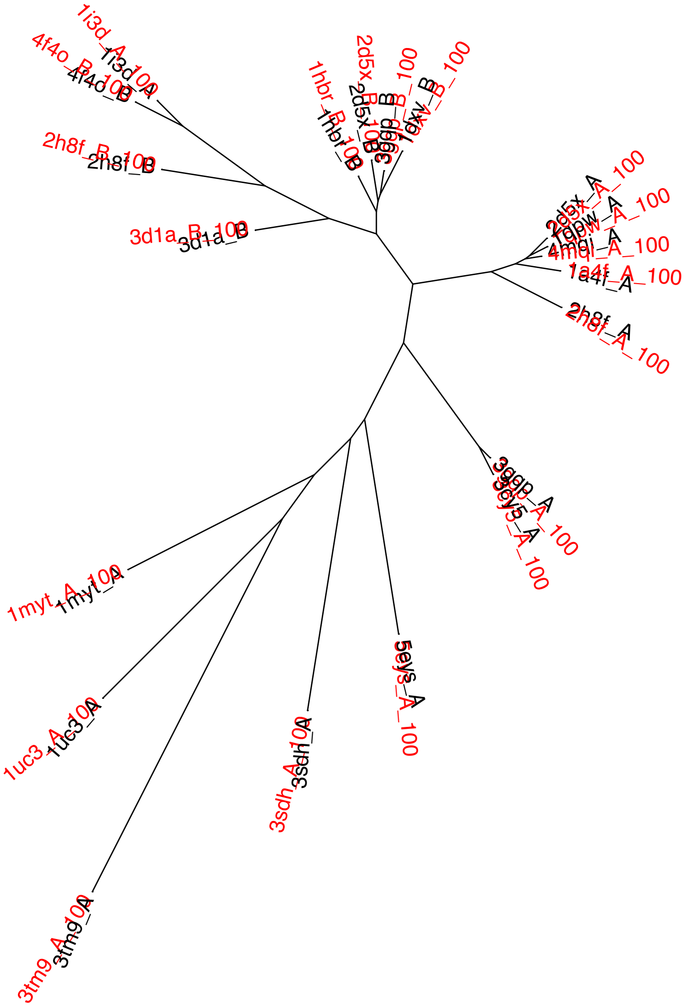

Figure S 12: Phylogenetic tree for proteins from the globin family built using structural datasets comprising a 100% of each structure together with the complete structures. The fractional structures are shown in red and the complete structures in black.

Trypsin-like serine proteases

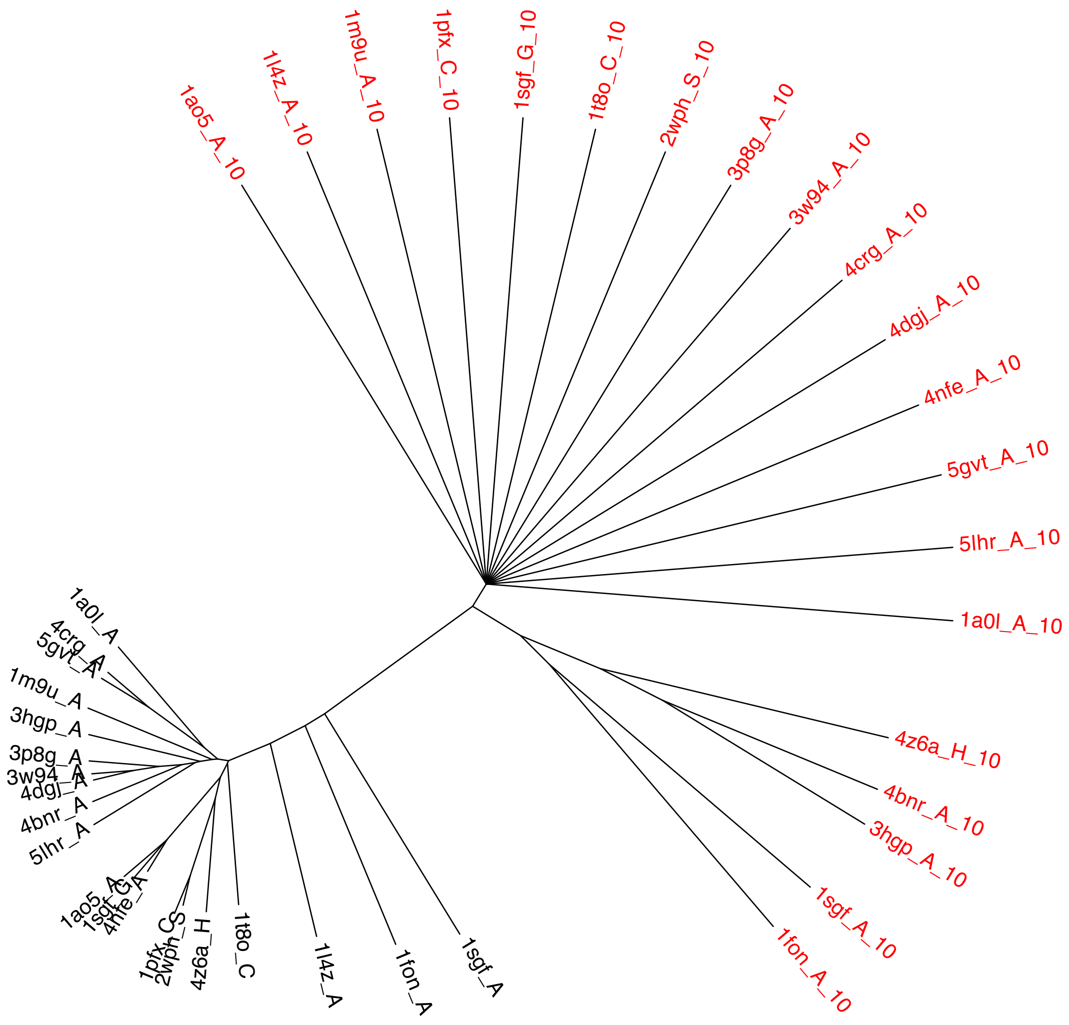

Figure S 13: Phylogenetic tree for proteins from the trypsin-like serine protease family built using structural datasets comprising a 10% of each structure together with the complete structures. The fractional structures are shown in red and the complete structures in black.

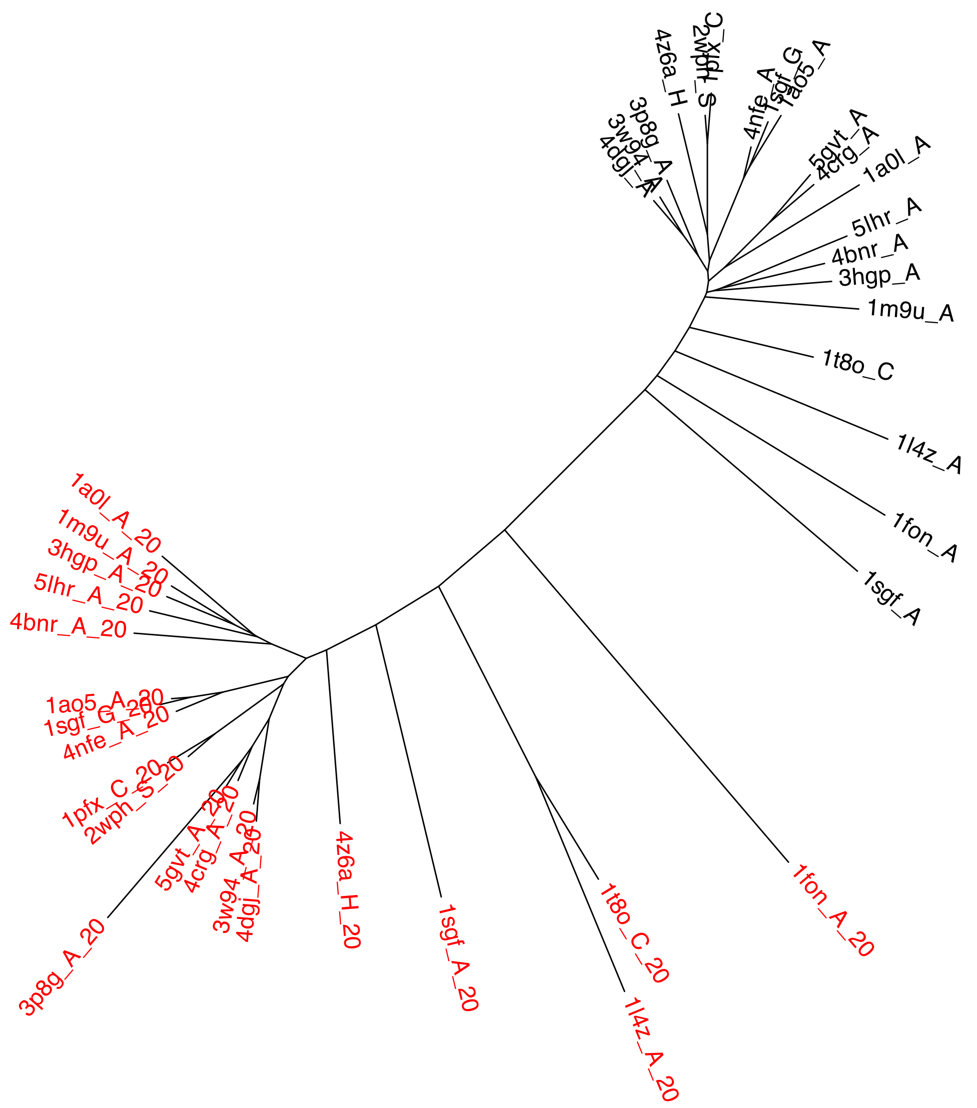

Figure S 14: Phylogenetic tree for proteins from the trypsin-like serine protease family built using structural datasets comprising a 20% of each structure together with the complete structures. The fractional structures are shown in red and the complete structures in black.

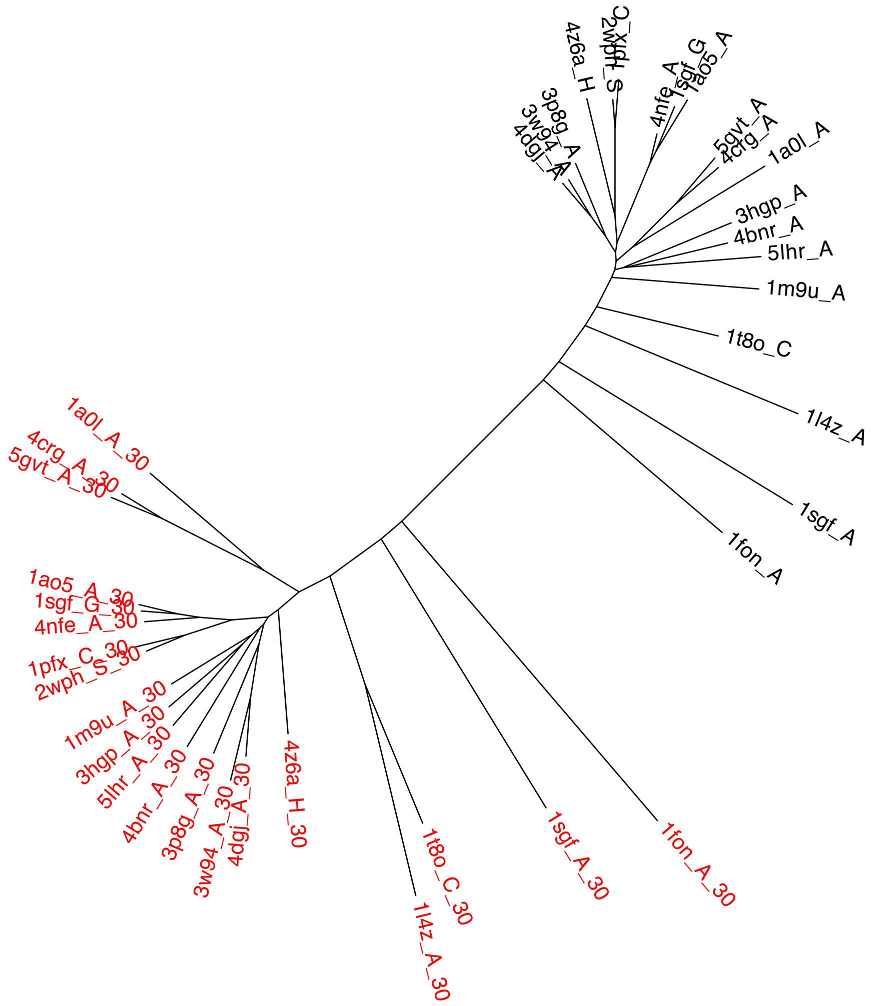

Figure S 15: Phylogenetic tree for proteins from the trypsin-like serine protease family built using structural datasets comprising a 30% of each structure together with the complete structures. The fractional structures are shown in red and the complete structures in black.

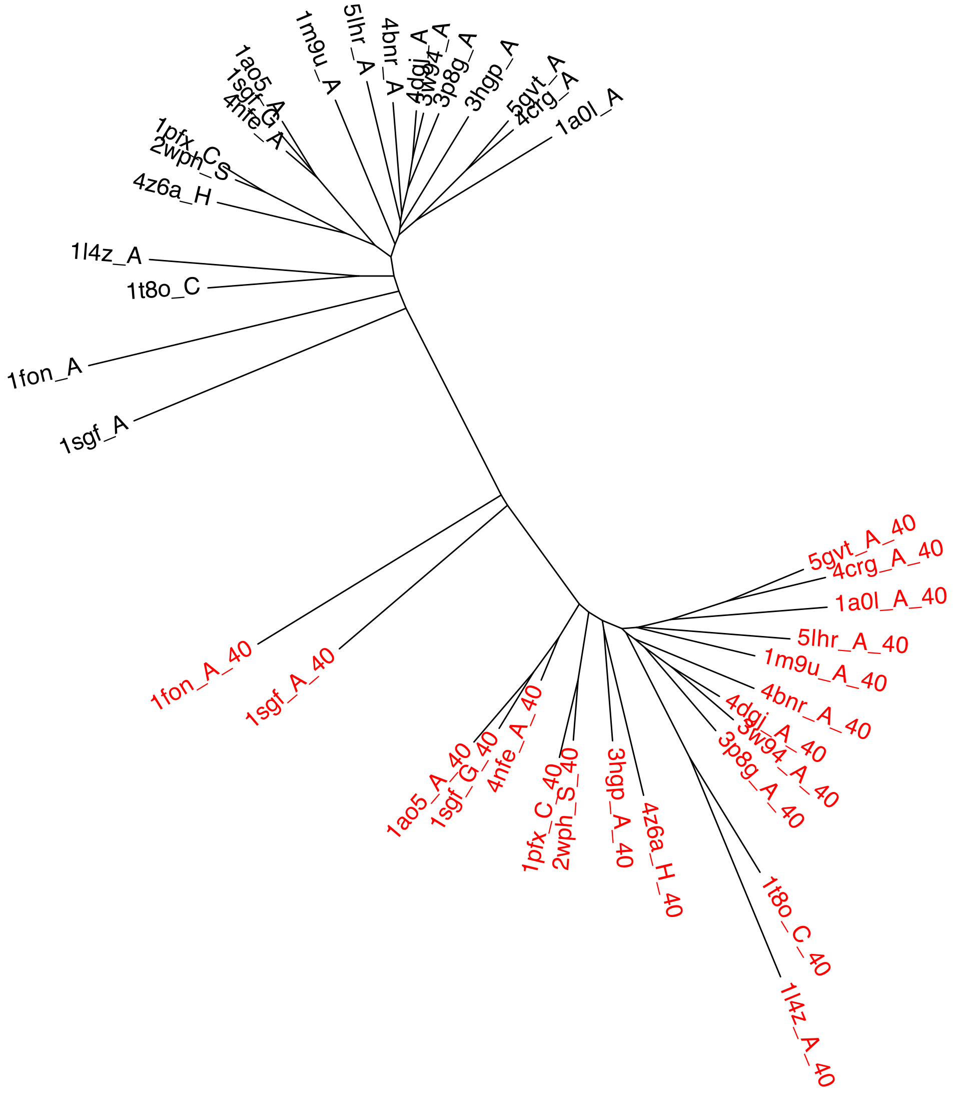

Figure S 16: Phylogenetic tree for proteins from the trypsin-like serine protease family built using structural datasets comprising a 40% of each structure together with the complete structures. The fractional structures are shown in red and the complete structures in black.

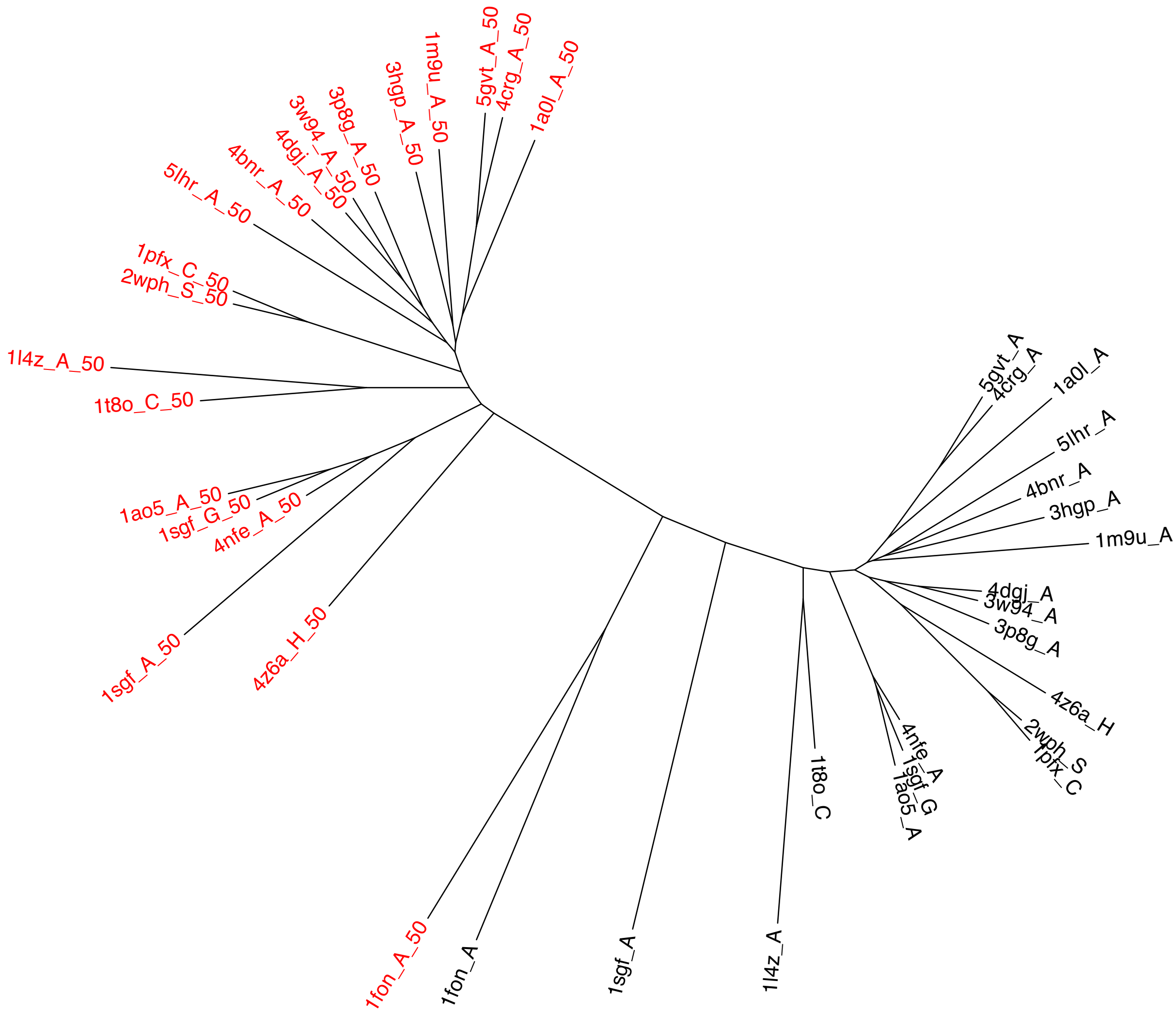

Figure S 17: Phylogenetic tree for proteins from the trypsin-like serine protease family built using structural datasets comprising a 50% of each structure together with the complete structures. The fractional structures are shown in red and the complete structures in black.

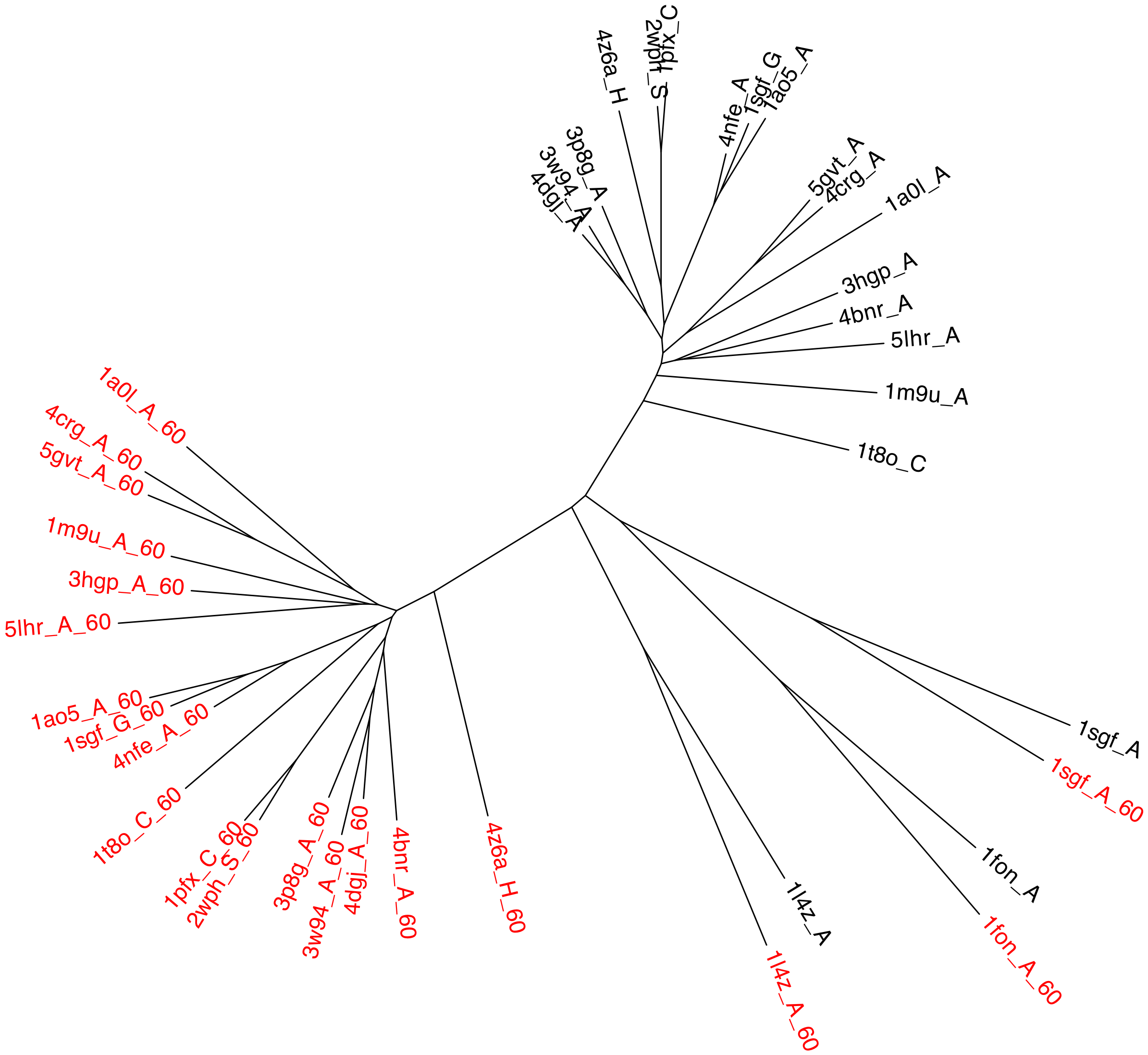

Figure S 18: Phylogenetic tree for proteins from the trypsin-like serine protease family built using structural datasets comprising a 60% of each structure together with the complete structures. The fractional structures are shown in red and the complete structures in black.

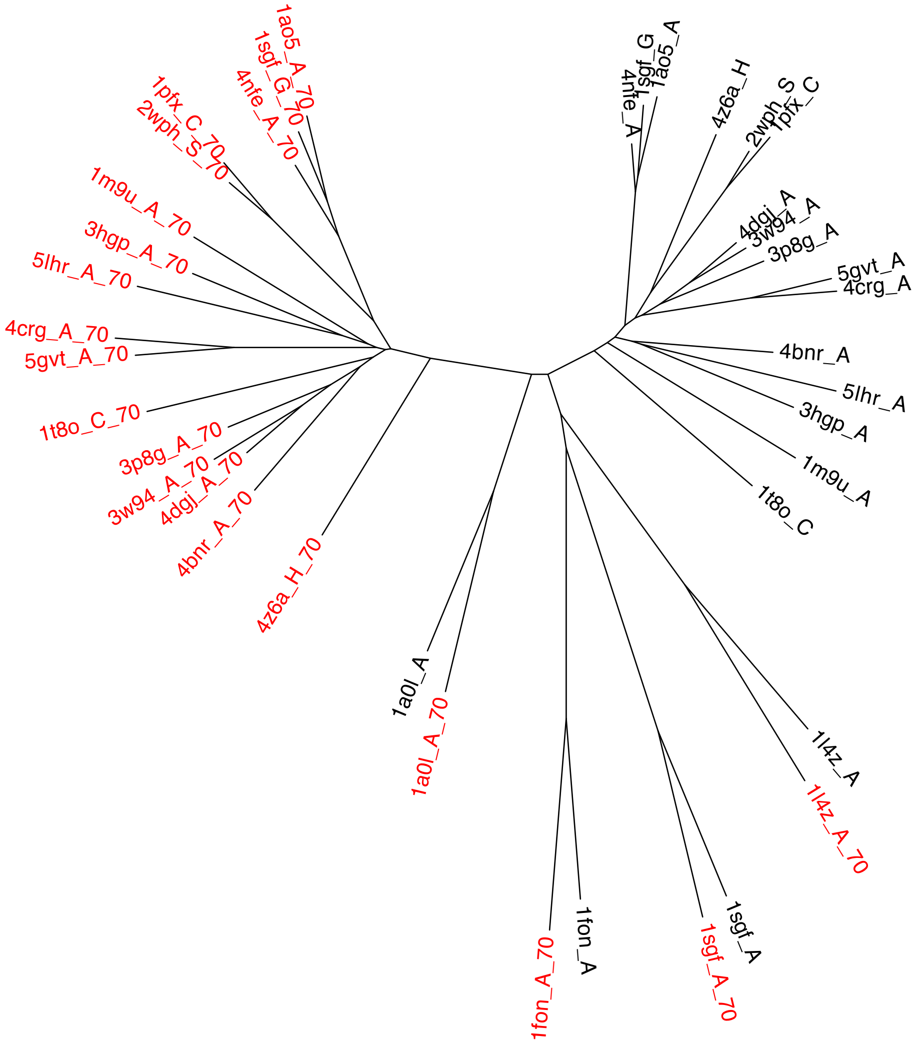

Figure S 19: Phylogenetic tree for proteins from the trypsin-like serine protease family built using structural datasets comprising a 70% of each structure together with the complete structures. The fractional structures are shown in red and the complete structures in black.

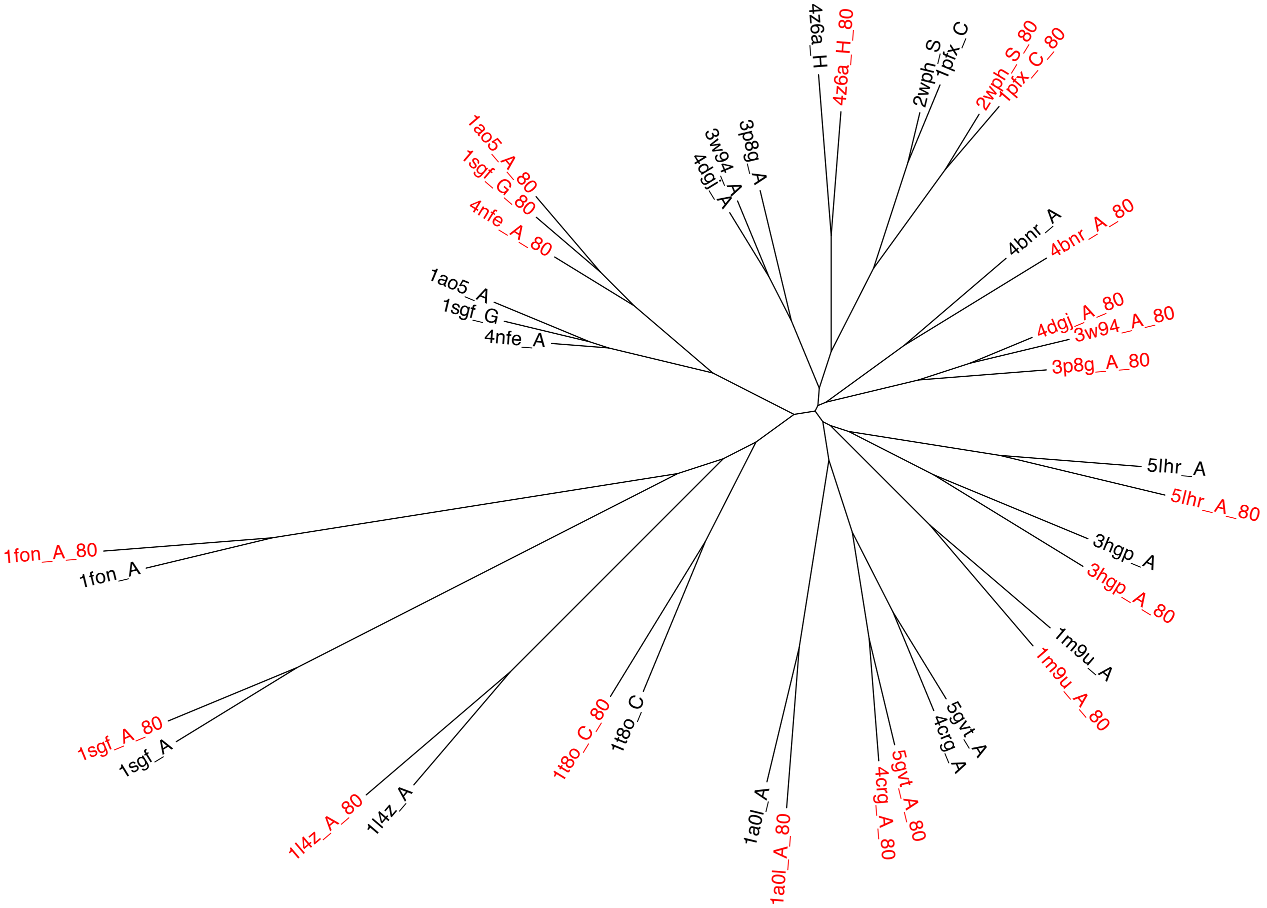

Figure S 20: Phylogenetic tree for proteins from the trypsin-like serine protease family built using structural datasets comprising a 80% of each structure together with the complete structures. The fractional structures are shown in red and the complete structures in black.

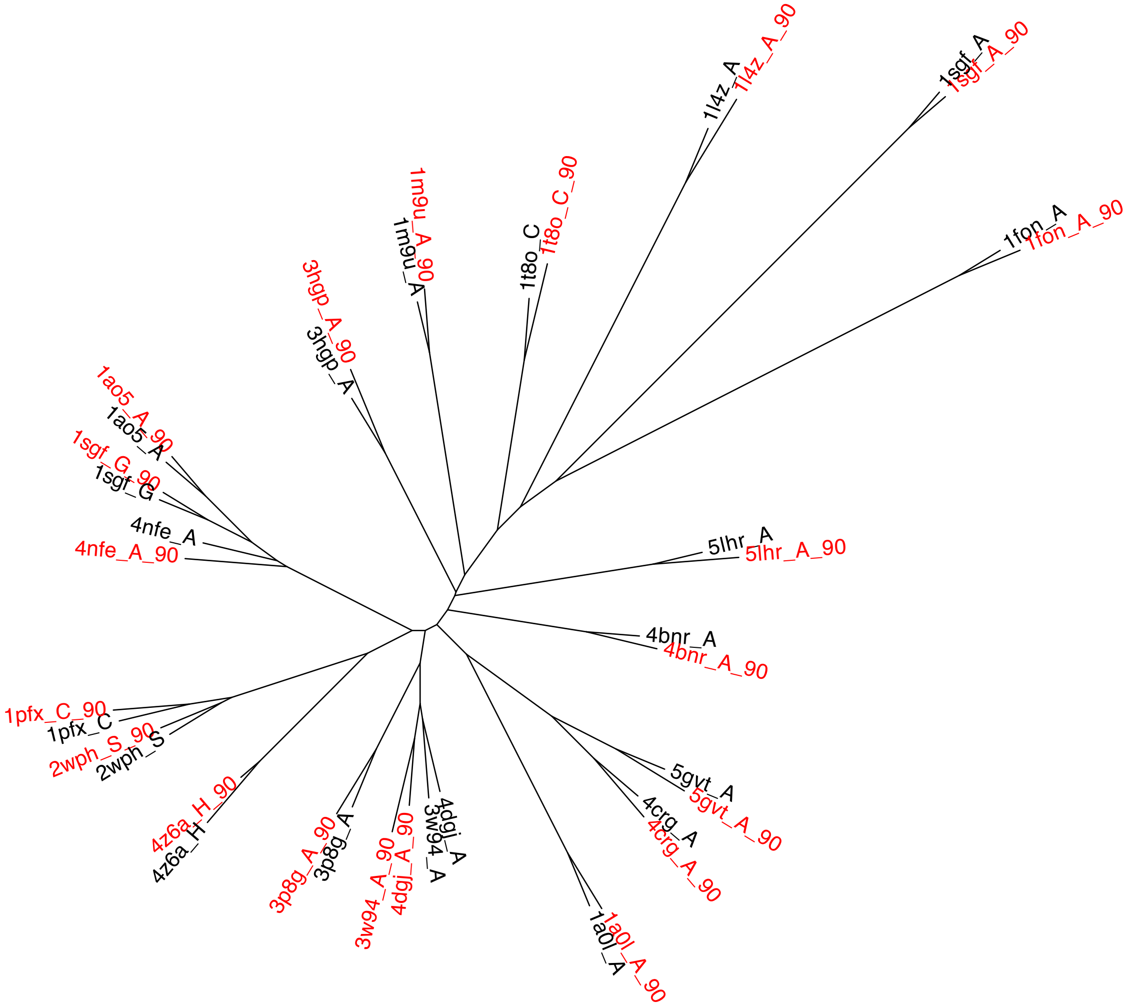

Figure S 21: Phylogenetic tree for proteins from the trypsin-like serine protease family built using structural datasets comprising a 90% of each structure together with the complete structures. The fractional structures are shown in red and the complete structures in black.

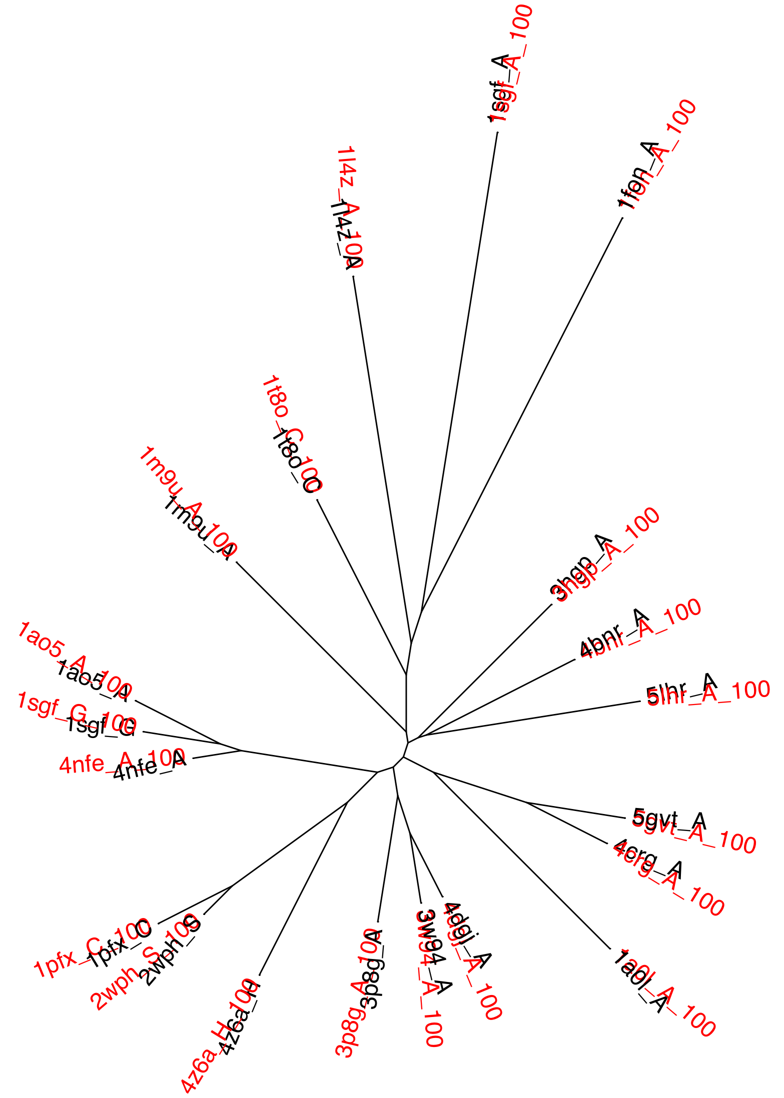

Figure S 22: Phylogenetic tree for proteins from the trypsin-like serine protease family built using structural datasets comprising a 100% of each structure together with the complete structures. The fractional structures are shown in red and the complete structures in black.

23 Aldo-keto reductase (NADP)

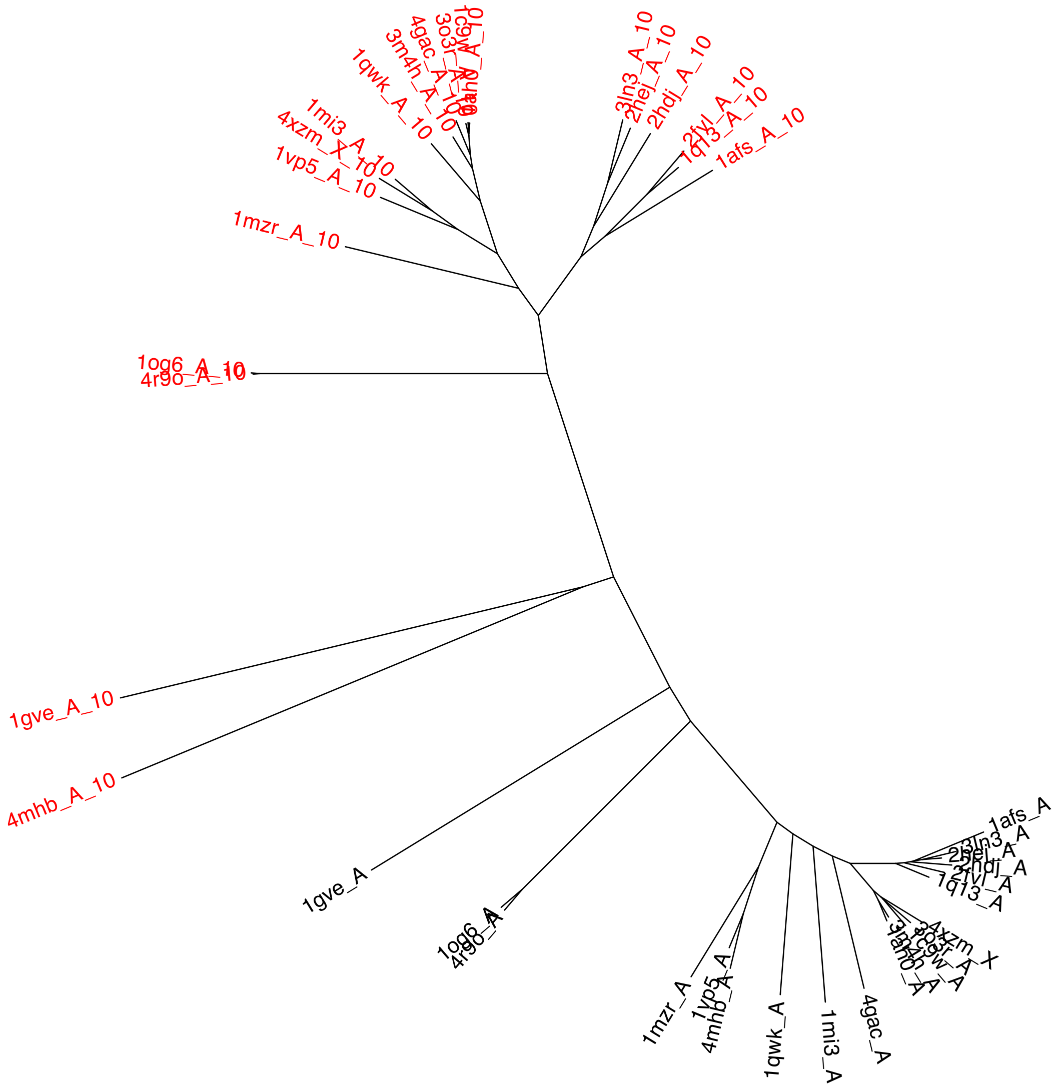

Figure S 23: Phylogenetic tree for proteins from the aldo-keto reductase (NADP) family built using structural datasets comprising a 10% of each structure together with the complete structures. The fractional structures are shown in red and the complete structures in black.

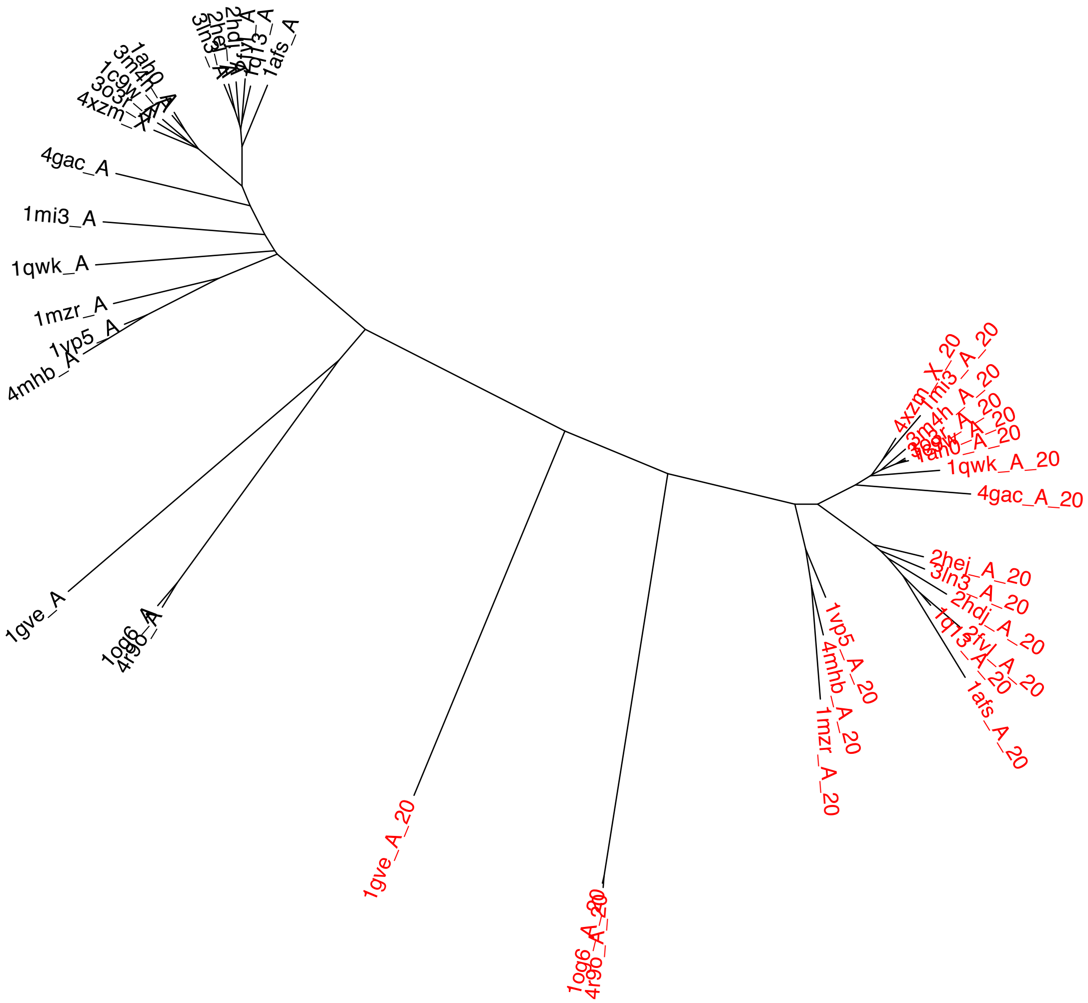

Figure S 24: Phylogenetic tree for proteins from the aldo-keto reductase (NADP) family built using structural datasets comprising a 20% of each structure together with the complete structures. The fractional structures are shown in red and the complete structures in black.

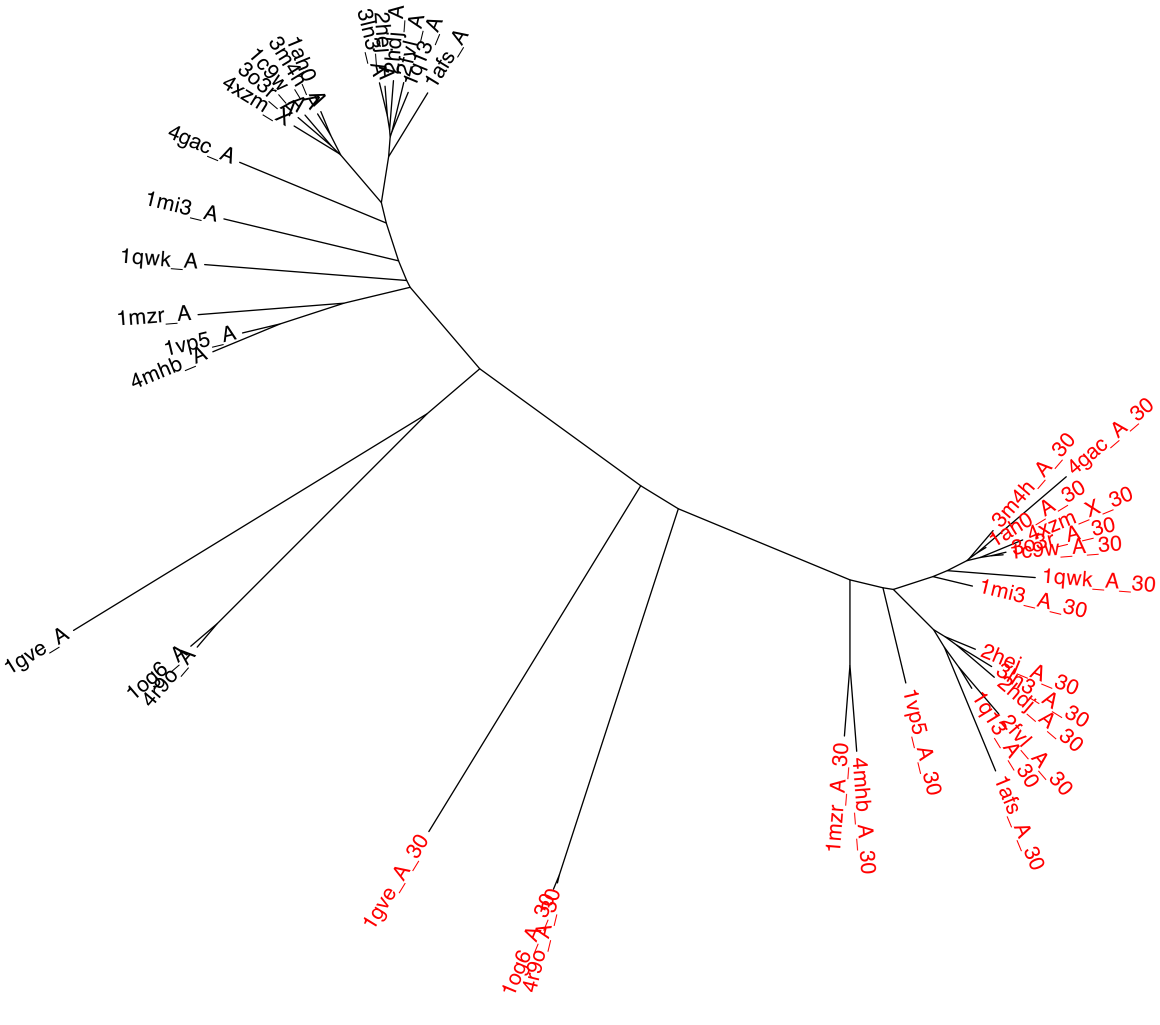

Figure S 25: Phylogenetic tree for proteins from the aldo-keto reductase (NADP) family built using structural datasets comprising a 30% of each structure together with the complete structures. The fractional structures are shown in red and the complete structures in blackk.

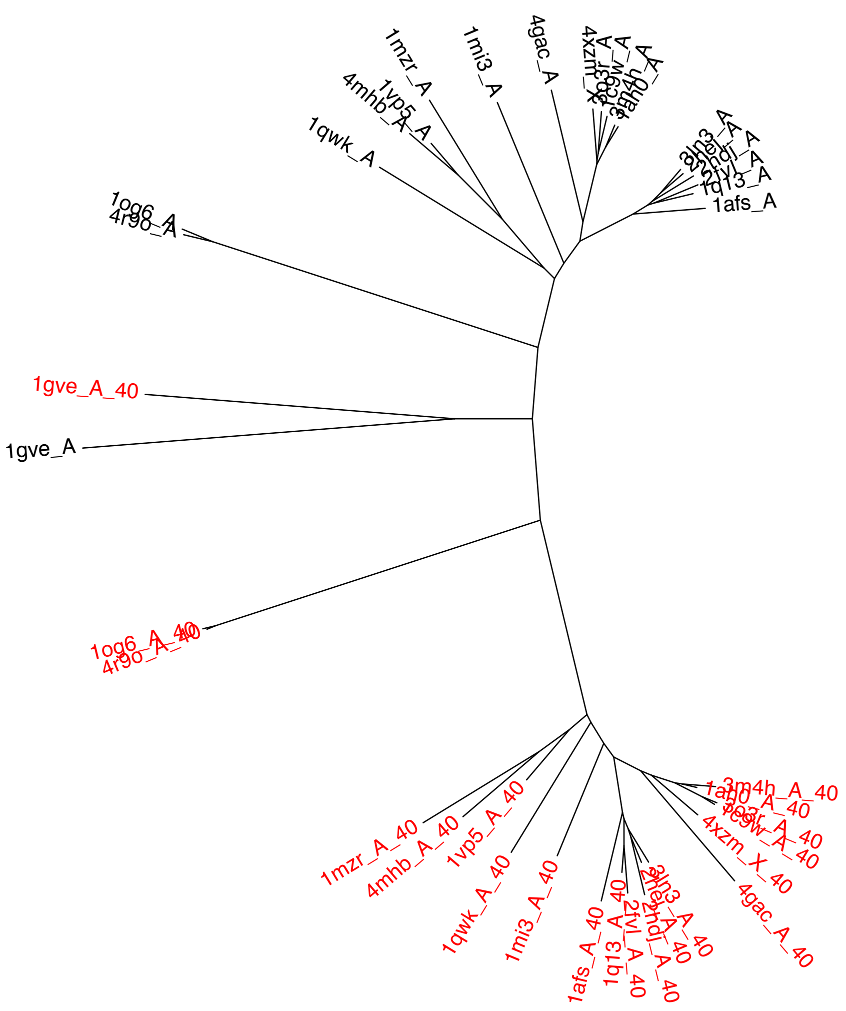

Figure S 26: Phylogenetic tree for proteins from the aldo-keto reductase (NADP) family built using structural datasets comprising a 40% of each structure together with the complete structures. The fractional structures are shown in red and the complete structures in black.

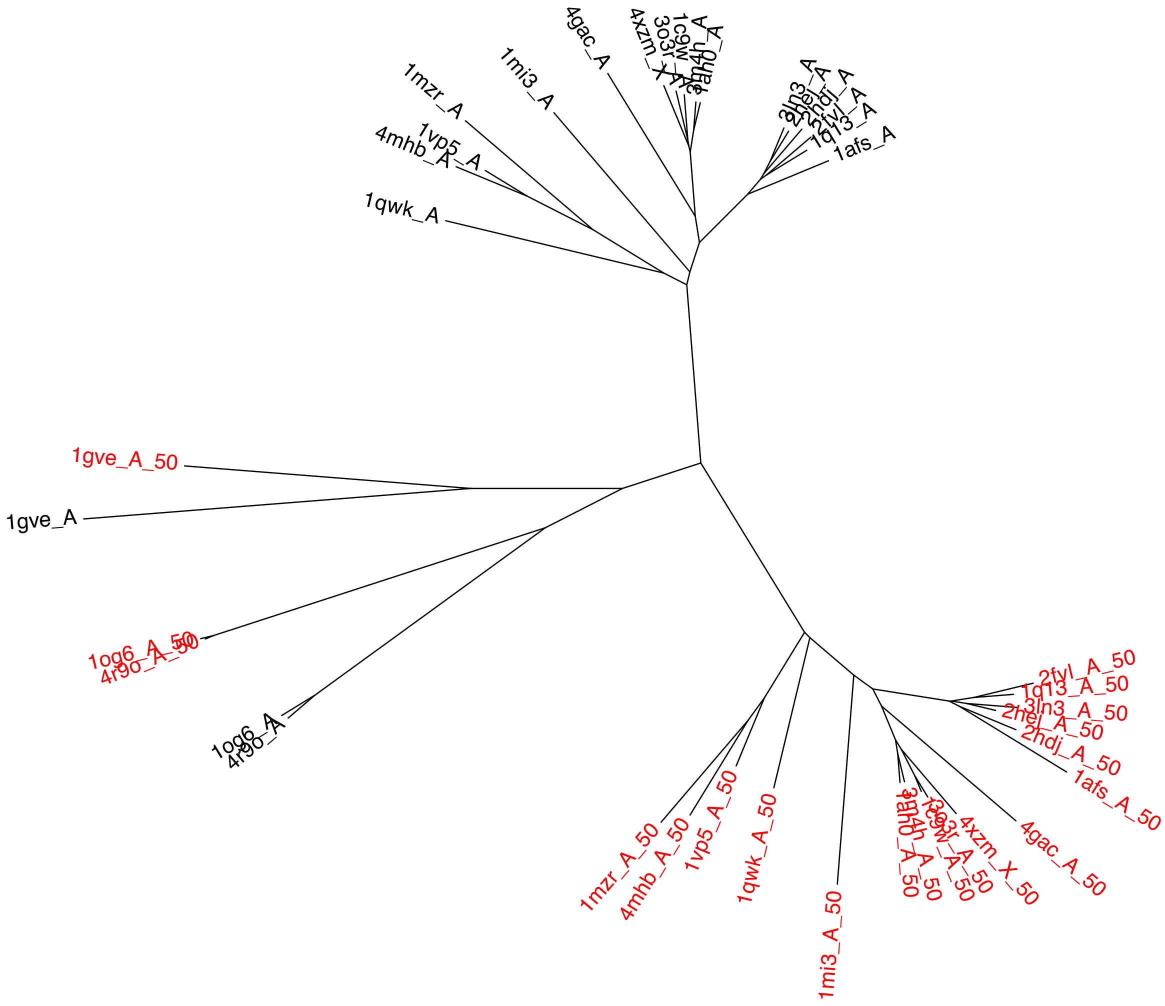

Figure S 27: Phylogenetic tree for proteins from the aldo-keto reductase (NADP) family built using structural datasets comprising a 50% of each structure together with the complete structures. The fractional structures are shown in red and the complete structures in black.

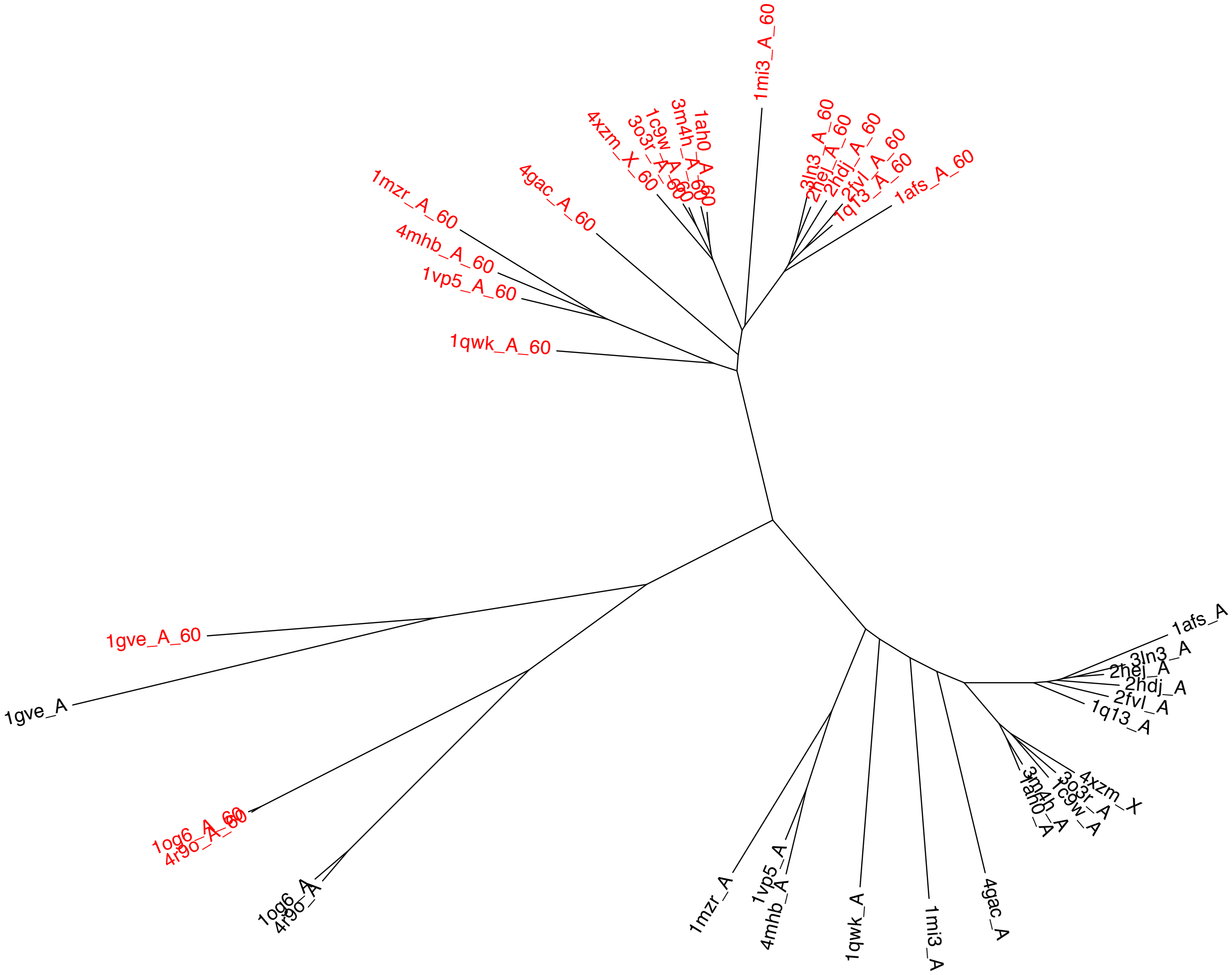

Figure S 28: Phylogenetic tree for proteins from the aldo-keto reductase (NADP) family built using structural datasets comprising a 60% of each structure together with the complete structures. The fractional structures are shown in red and the complete structures in black.

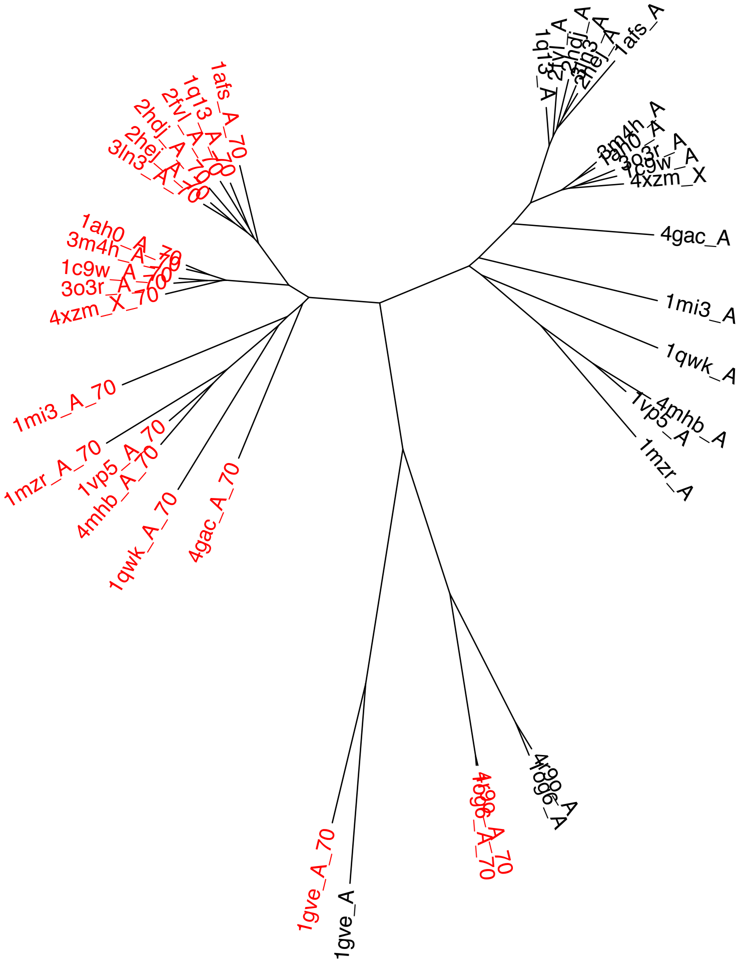

Figure S 29: Phylogenetic tree for proteins from the aldo-keto reductase (NADP) family built using structural datasets comprising a 70% of each structure together with the complete structures. The fractional structures are shown in red and the complete structures in black.

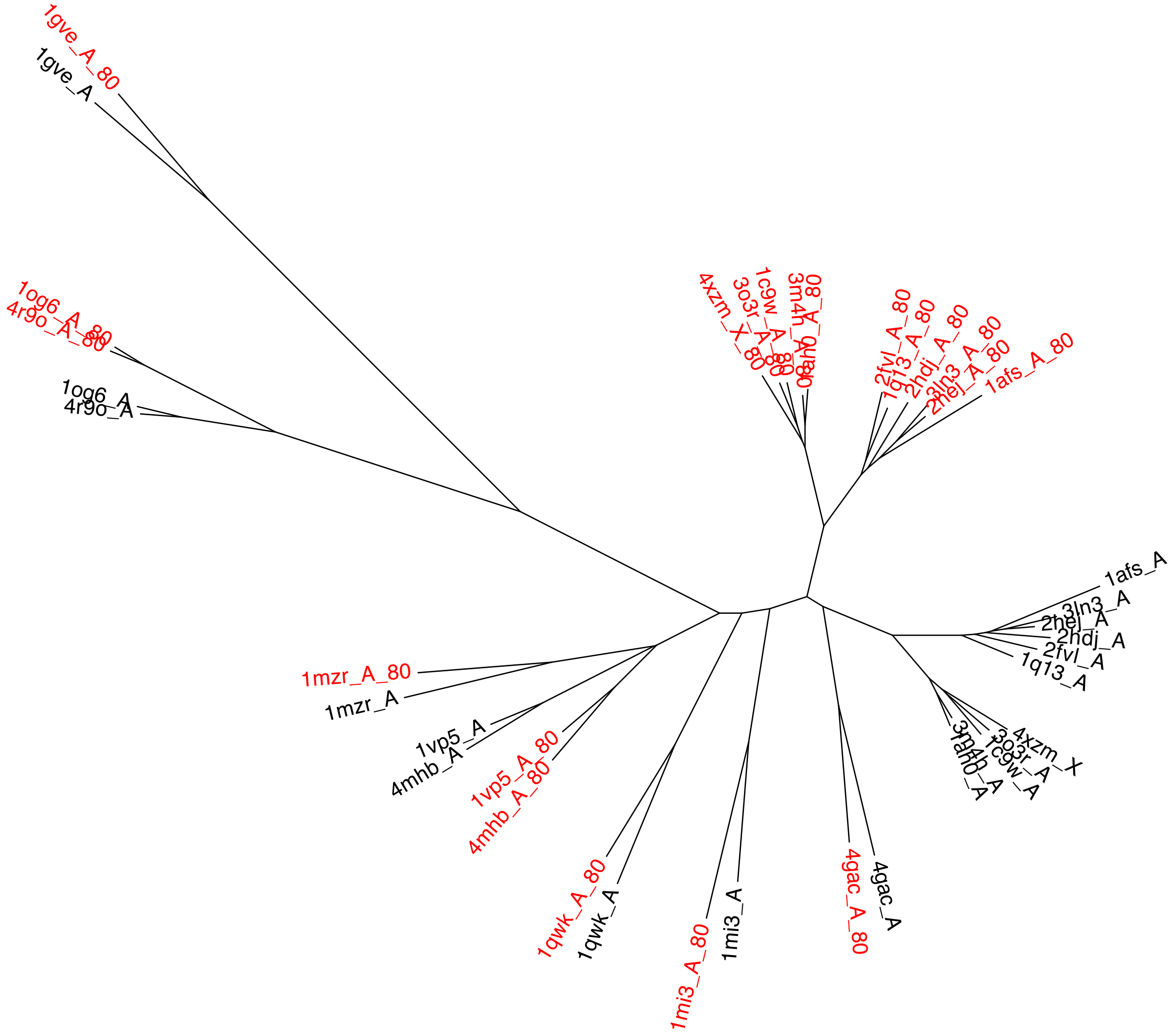

Figure S 30: Phylogenetic tree for proteins from the aldo-keto reductase (NADP) family built using structural datasets comprising a 80% of each structure together with the complete structures. The fractional structures are shown in red and the complete structures in black.

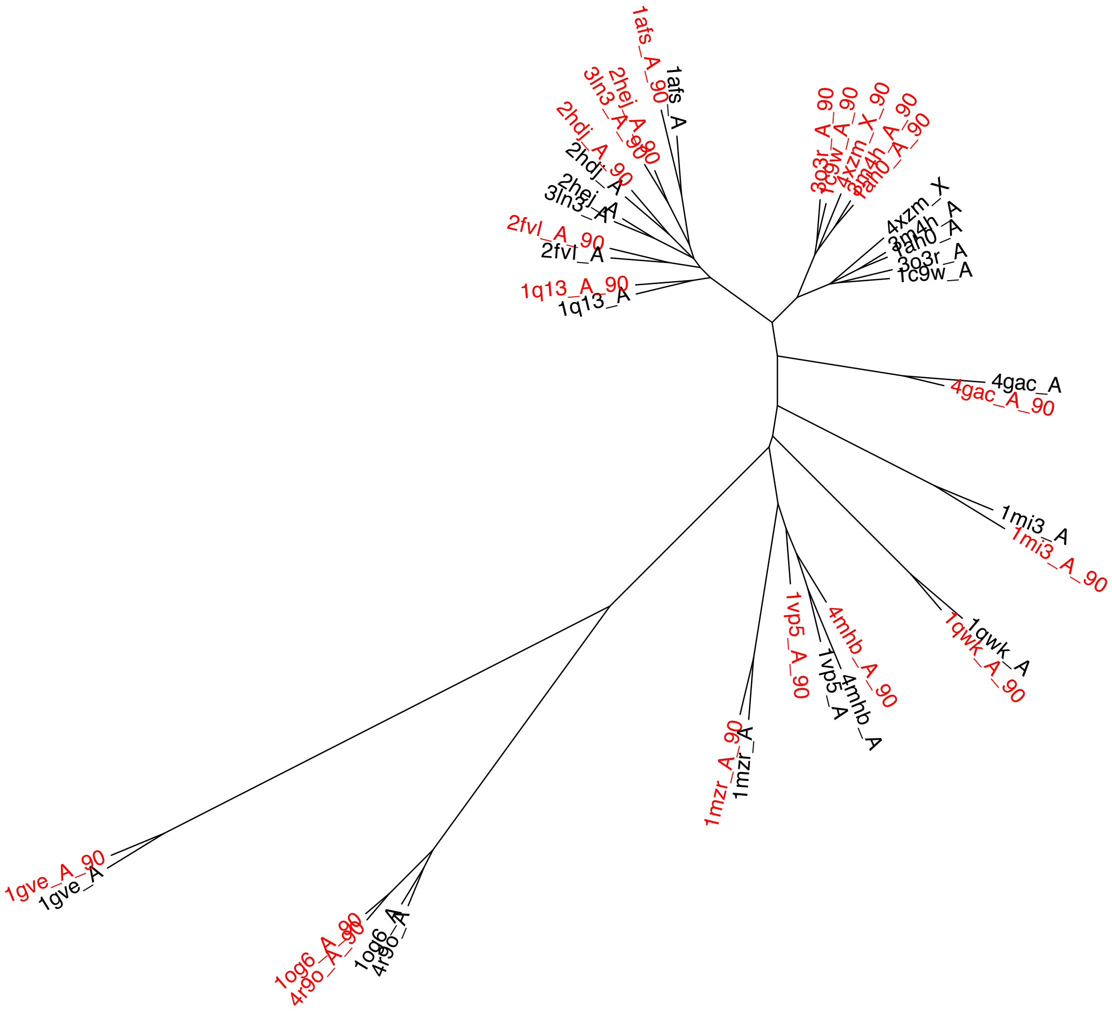

Figure S 31: Phylogenetic tree for proteins from the aldo-keto reductase (NADP) family built using structural datasets comprising a 90% of each structure together with the complete structures. The fractional structures are shown in red and the complete structures in black.

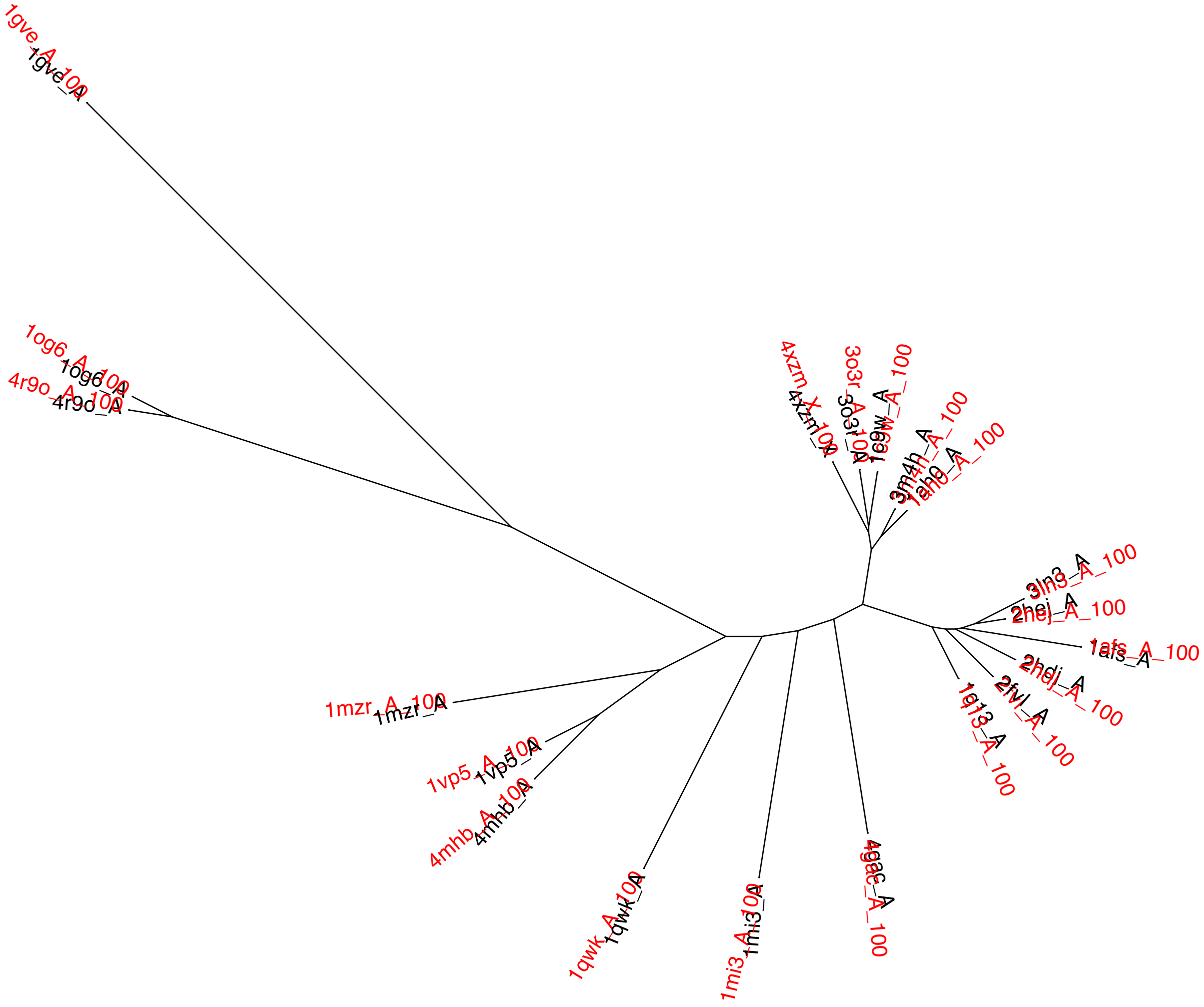

Figure S 32: Phylogenetic tree for proteins from the aldo-keto reductase (NADP) family built using structural datasets comprising a 100% of each structure together with the complete structures. The fractional structures are shown in red and the complete structures in black.

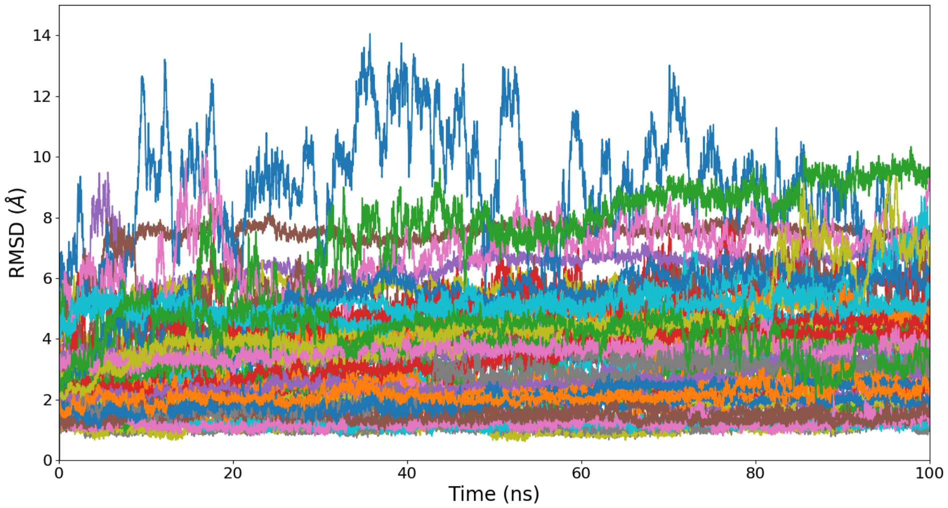

(a)

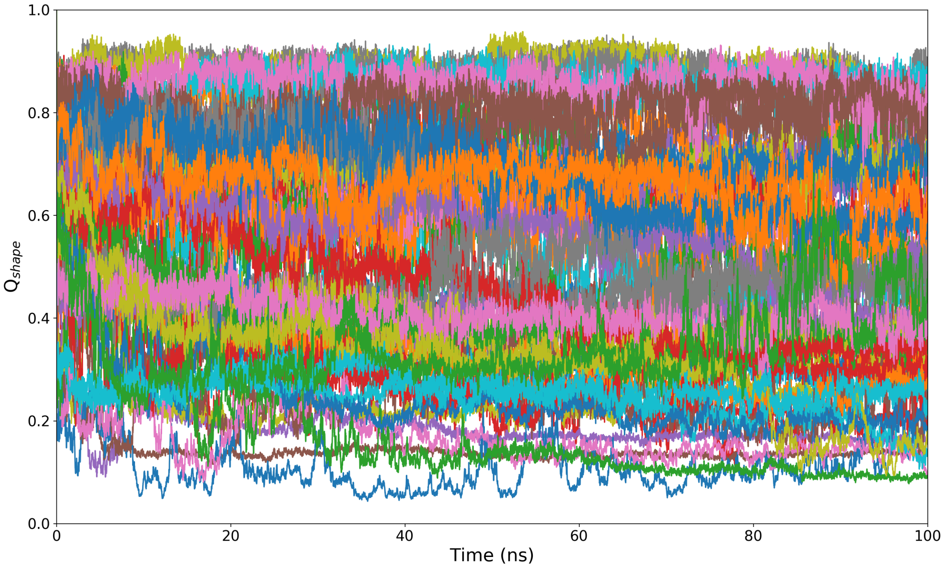

(b)

Figure S 33: Change in the a) Cα atom-positional RMSD from the starting conformation and b) Q_{shape} of each of 53 protein structures from the ferritin-like superfamily during MD simulations.

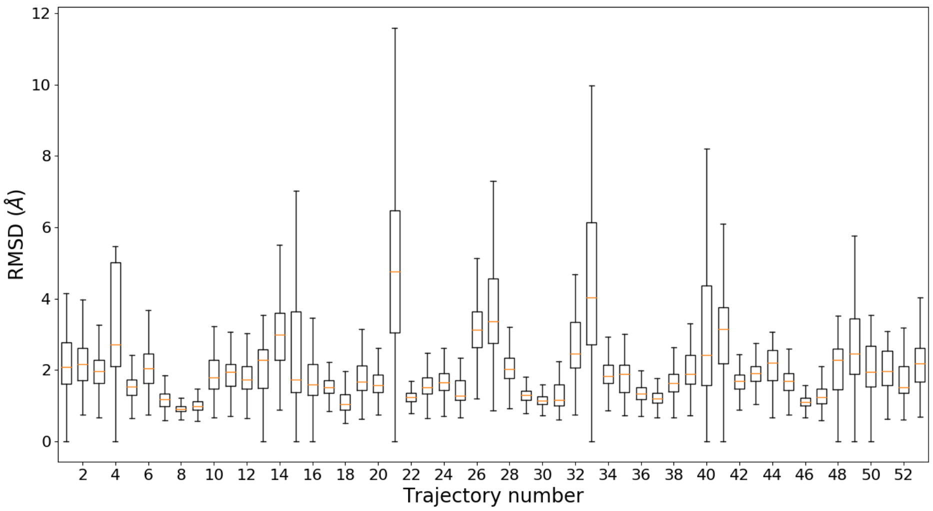

Figure S 34: The range of values of the Cα atom-positional RMSD from the central structure of the simulated ensemble of each of 53 protein structures from the ferritin-like family.

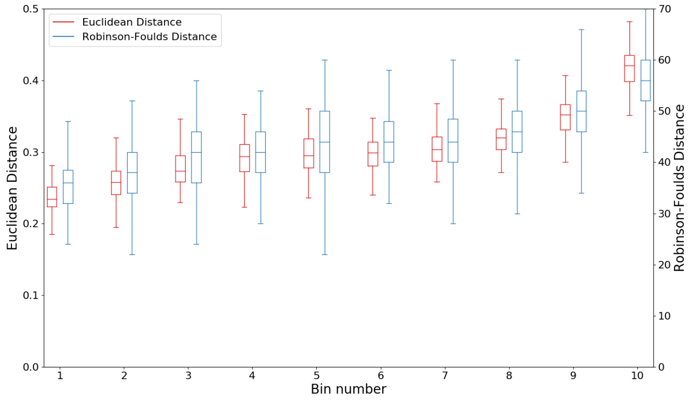

(a)

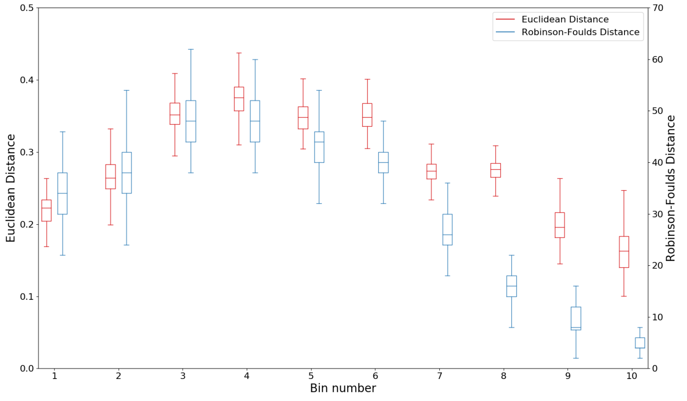

(b)

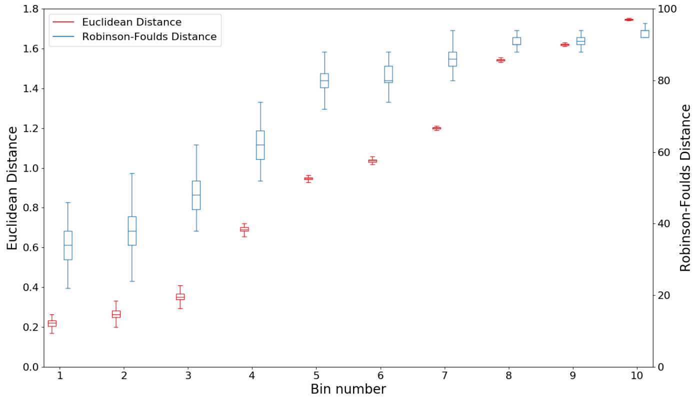

(c)

Figure S 35: The range of Euclidean and Robinson-Foulds(Robinson and Foulds, 1981) distances between the reference tree and 1000 sample trees from each bin of (a) ranked RMSD values and (b) and (c) raw RMSD values with respect to the central structure of the simulated ensemble of each of 50 proteins from the ferritin-like superfamily. In (a) and (c), as the bin number increases and (non-uniformly in (a)) the RMSD values increase, the topologies of the reference and sample trees diverge. In (b), the trees appear to become more similar as bin number increases, likely due to the decreasing number of leaves of the tree.

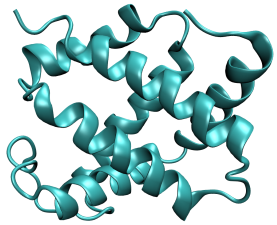

(a) 1gcv_A

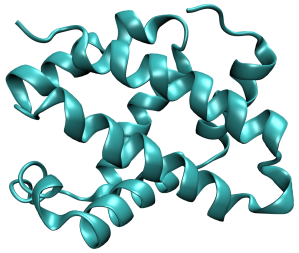

(b) 1gcv_B

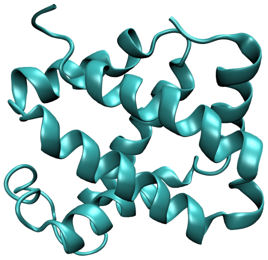

(c) 1hv4_A

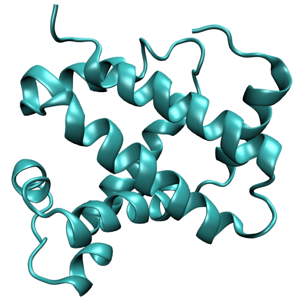

(d) 1hv4_B

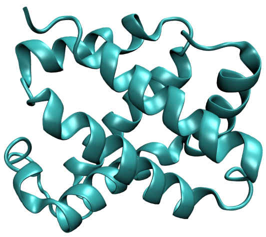

(e) 2dn2_A

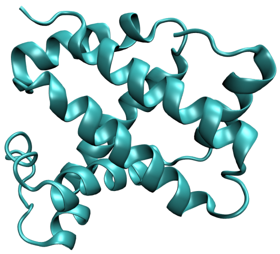

(f) 2dn2_B

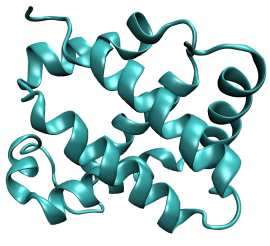

(g) 3hrw_A

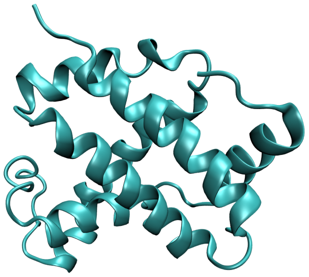

(h) 3hrw_B

Figure S 36: Structures of four α- and four β-haemoglobin chains, highlighting their high degree of structural conservation.

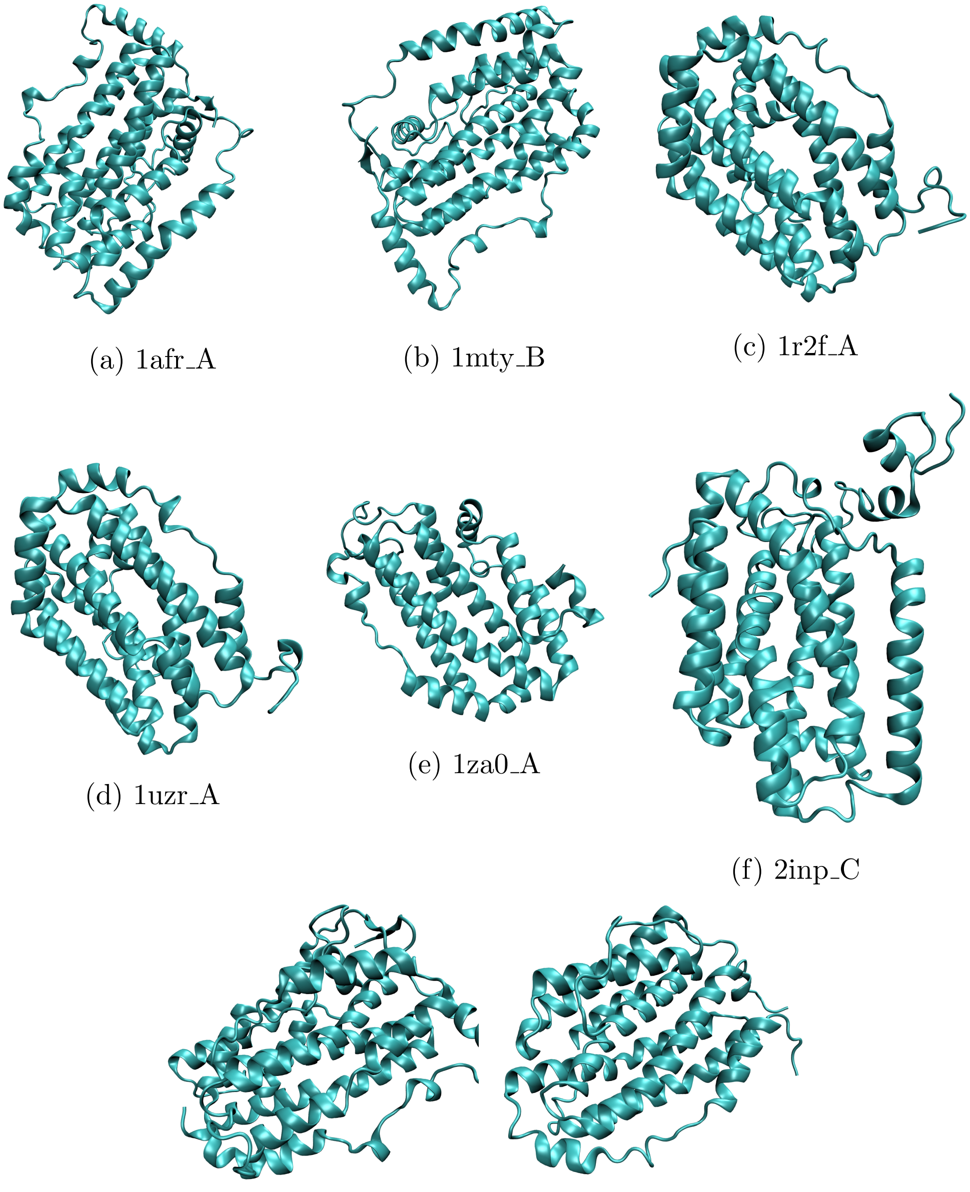

(a) 1afr_A (b) 1mty_B (c) 1r2f_A

(d) 1uzr_A (e) 1za0_A (f) 2inp_C

(g) 2uw1_A (h) 2uw2_B

Figure S 37: Structures of eight ribonucleotide reductase-like proteins, which show more structural divergence compared to the α- and β-haemoglobins.

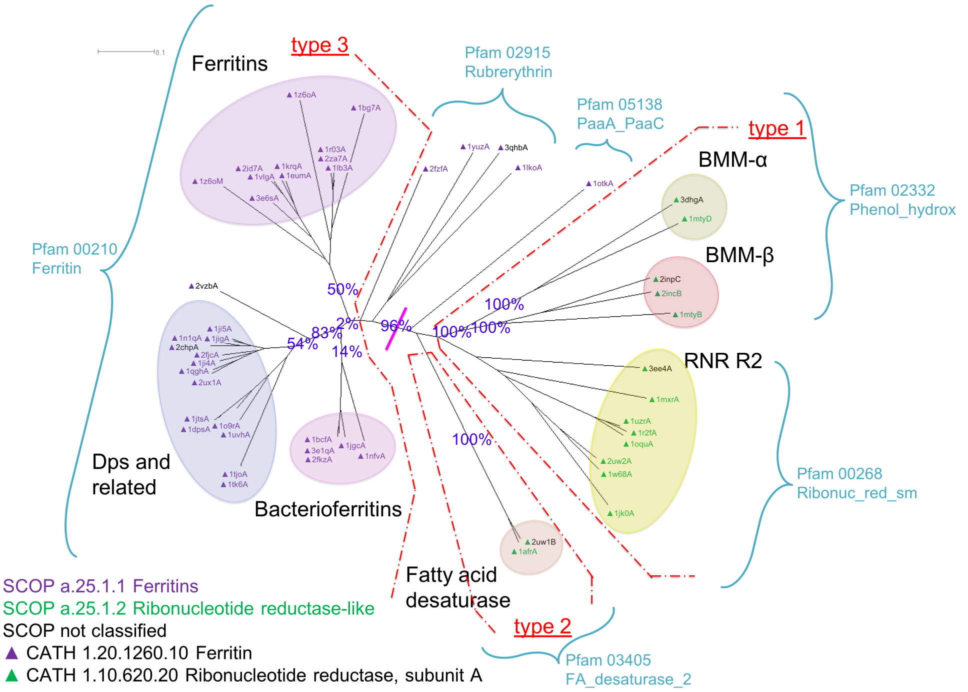

(a)

Figure S 38: Structural phylogeny of the ferritin-like superfamily with statistical support from MC sampling and the structural bootstrap method. The colour-coded ellipses are consistent with the previous study (Lundin et al., 2012) and labelled with annotations provided by the RCSB PDB [REF]. The scale bars represent distance as quantified by the inverse Q_{score}. The bifurcating tree was built using the structures from which the simulations were initiated, with statistical support generated using MC simulations. Support values obtained from 100 samples of alternative conformations for each protein structure from the repertoire of 2,500 conformations generated during the production phase of the MC simulation are shown for key splits. SCOP and CATH classifications are shown by the colour of the node labels and of the associated triangle, respectively, as per the embedded key. Pfam classifications are indicated by arcs.

References

Lundin D, Poole AM, Sjöberg BM, and Högbom M. 2012. Use of structural phylogenetic networks for classification of the ferritin-like superfamily. *Journal of Biological Chemistry*, 287(24):20565–20575.

Robinson D and Foulds L. 1981. Comparison of phylogenetic trees. *Mathematical Biosciences*, 53(1):131 – 147.

wwPDB consortium. 10 2018. Protein Data Bank: the single global archive for 3D macromolecular structure data. *Nucleic Acids Research*, 47(D1): D520–D528. ISSN 0305-1048. doi: 10.1093/nar/gky949. URL `https://doi.org/10.1093/nar/gky949`.
